# Preparedness, awareness and ownership of young future healthcare professionals are associated with their willingness to contribute to health system resilience in a NATO collective defense scenario: a cross-sectional study among medical, nursing and paramedic students in Germany

**DOI:** 10.1186/s12889-026-29675-1

**Published:** 2026-10-02

**Authors:** Lara Amling, Isabella Beyer, Felix Hambitzer, Nico Neu, Jonas Rickermann, Johannes Weidt, Timo Ulrichs

**Affiliations:** 1 RWTH Aachen University, Aachen, Germany; 2 Department of Neurology, University Hospital Regensburg, Regensburg, Germany; 3 Charité – University Medicine Berlin, Berlin, Germany; 4 University of Tübingen, Tübingen, Germany; 5 Bielefeld University, Bielefeld, Germany; 6 University of Oxford, Oxford, England; 7 Akkon University of Applied Sciences, Berlin, Germany

**Keywords:** NATO collective defense, Disaster preparedness, Ownership, Willingness, Awareness, Health system resilience

## Abstract

**Background:**

Russia’s war against Ukraine has renewed discussion about a possible NATO Article 5 collective defense scenario in Europe. In such a situation, Germany would function as key logistical and medical hub, placing substantial strain on its healthcare system. Experience from the COVID-19 pandemic suggests that health system resilience not only depends on infrastructure but also on availability and commitment of health professionals. This study examined willingness of future health professionals in Germany to contribute beyond routine duties in such a crisis and assessed preparedness, ownership, and awareness as potential determinants.

**Methods:**

Between 20 May and 15 July 2025, a cross-sectional online survey was conducted among German medical, nursing, and paramedic students (*n* = 514). Data were collected on demographics and self-assessed individual preparedness, perceived institutional preparedness, voluntary engagement as major indicator of ownership, and awareness regarding a NATO collective defense scenario. Willingness to contribute beyond routine practice was assessed across preparatory activities, crisis-specific medical tasks, and modified working conditions. Composite scores were calculated for awareness, individual and institutional preparedness, and willingness. Associations with willingness were examined using multivariable linear regression adjusted for predefined covariates.

**Results:**

Overall, 48.8% considered a NATO collective defense scenario by 2030 likely, 83.3% anticipated negative effects on the German healthcare system, and 94.5% perceived the healthcare system as poorly prepared. Individual preparedness was low (median: 3 on 0–12 scale; IQR: 1–5), while recent voluntary engagement, including voluntary work during studies or apprenticeships in general or in civil aid organizations specifically, was common (59.1%). Participants showed high willingness to engage in preparatory measures and to provide care in Germany, but lower willingness to initiate trainings or to provide care for German or allied soldiers abroad. In adjusted regression analyses, higher perceived institutional preparedness, voluntary engagement, and awareness were significantly associated with greater willingness (all: *p* < 0.01).

**Conclusion:**

Willingness of future health professionals to support the German healthcare system in a NATO collective defense scenario was associated with perceived institutional preparedness, awareness, and voluntary engagement. Widespread perceptions of insufficient preparedness raise concerns about system resilience, and the associated factors might represent potential targets for policy interventions.

**Supplementary Information:**

The online version contains supplementary material available at https://doi.org/10.1186/s12889-026-29675-1 .

## Background

### Political situation

The geopolitical situation has changed markedly in recent years, not least because of Russia’s war against Ukraine [1]. These developments have prompted renewed evaluations of the global security environment. Experts are increasingly considering the possibility of an attack on North Atlantic Treaty Organization (NATO) territory along the alliance’s eastern flank in the foreseeable future [2–4]. Such an attack could invoke Article 5 of the North Atlantic Treaty [5]. Article 5 is the core provision and regulates the collective defense mechanism of the NATO member states, obliging them to support one another in the event of an attack [5].

In this scenario, Germany would assume a central role due to its geographical location and its function as a strategic hub for NATO’s Host Nation Support (HNS) [6]. According to the German Armed Forces [6], the HNS provides assistance to allied troops in seven areas - including healthcare - while they are in transit through Germany during both the deployment and redeployment phases. Hence, wounded Allied and German forces would receive treatment in Germany, creating substantial demand for medical care. It is unlikely that military medical services alone will be capable of meeting such a demand, given the estimated number of approximately 1000 wounded German soldiers per day as additional patients and the simultaneous deployment of military medical personnel to conflict areas to provide direct on-site support [7, 8]. Consequently, civilian healthcare structures would inevitably be involved in managing the unprecedented care demands associated with a major conflict scenario. This situation has occurred in the Ukrainian healthcare system following Russia’s full-scale invasion in 2022 [9]. Accordingly, the resilience of the health system constitutes a key component of overall system resilience [10].

### Health system resilience

The resilience of the German civil health system to withstand such a scenario needs a thorough evaluation, particularly considering the experiences gained from previous crises, such as the COVID-19 pandemic [10]. Prior to the pandemic, the health system was grappling with significant staffing shortages, and emergency preparedness plans were either incomplete or insufficiently tested [11–13]. The substantial influx of complex patients, together with temporary shortages of protective equipment and personnel during the pandemic, placed significant pressure on the healthcare system and exposed its resilience deficiencies [10, 12].

The concept of health system resilience provides a useful framework for analyzing these challenges and for identifying factors that may enhance Germany’s capacity to fulfill its responsibilities within a NATO collective defense scenario.

According to Lega and Castellini [14], health system resilience is defined as “the capacity of a system to prepare for and respond to a health crisis and threats, maintain its core function during crises, learn from crises, and transform its setting based on the acquired lessons, where crises include sudden and unexpected health shocks and chronic and predictable health threats”. Within this framework, human resources represent an important component of health system resilience. In the event of a NATO collective defense, the contributions of physicians, nurses and paramedics would be indispensable to ensure continuity of care under crisis conditions [15].

### Role of young health care professionals

As our study assesses future health system resilience, we focused on current medical students as well as nursing and paramedical trainees, who will constitute the workforce in the coming decade. The relevance of this group was highlighted during the COVID-19 pandemic, when students were mobilized to support intensive care units, vaccination campaigns, and public health authorities, thereby directly contributing to the healthcare delivery in a time of acute stress [16].

In Germany, medical students complete a direct-entry university programme of at least six years [17], whereas nursing students [18, 19] and paramedic trainees [20, 21] traditionally undergo three years of vocational training, with a primary-qualifying Bachelor’s degree available for nursing as an alternative since 2020 [22].

Preparing future healthcare professionals for large-scale crises requires competencies beyond routine clinical care. In Germany, a recommended curriculum for disaster medicine in medical education defines essential competencies for managing large-scale emergencies [23, 24], that are also relevant in military conflict scenarios. However, as implementation of this curriculum remains voluntary, most medical faculties offer disaster medicine only as an elective or extracurricular activity, and only a minority have established it as a mandatory, dedicated subject [25]. In light of the current geopolitical situation, disaster medicine has recently been added as an elective to the curriculum of several further medical schools [26] and a teaching module on disaster nursing has recently been developed [27].

Assessing future healthcare professionals’ familiarity with these disaster medicine–specific and other emergency preparedness competencies is therefore important when considering their contribution to health system resilience under extraordinary circumstances.

### Measuring health system resilience and its influencing factors

To assess health system resilience in crisis scenarios, it is essential to understand whether healthcare professionals would be willing to assume increased workloads and altered work environments, and which factors determine this willingness. The possession of a healthcare profession does not inherently guarantee readiness to surpass conventional obligations under extraordinary circumstances such as a military conflict [28]. Rather, the literature suggests that willingness is influenced by multiple factors. In their qualitative study of international healthcare workers during the 2014–2016 West African Ebola outbreak, Gee and Skovdal [29] identify institutional trust, past experience, and the sense of self-efficacy as important attenuators of perceived risk. A qualitative study on healthcare workers’ attitudes to working during an influenza pandemic in the UK similarly describes willingness as shaped by a sense of obligation to work, but also by barriers such as lack of information, unclear role expectations, limited trust in the national healthcare system, and concerns for family wellbeing [28]. Complementing these findings, a report by the German Parliament emphasizes the importance of awareness of potential crises and of responsibility across system levels [10].

These recurring determinants, threat perception, preparedness, and self-efficacy, are also inherent to established behavioral frameworks, most notably the Protection Motivation Theory [30] and the Health Belief Model [31], which conceptualize protective behavior as a function of how individuals assess a threat and their capacity to respond to it. A systematic review by Nafar et al. [32] supports these constructs as recurring predictors of healthcare workers’ willingness to respond in disaster contexts. However, willingness in a collective defense scenario is not solely an individual decision but is also embedded in social and institutional structures, with health system resilience depending on both organizational capacity and the prosocial engagement of those working within it [33].

### Introduction of the POWA framework

**Fig. 1:**
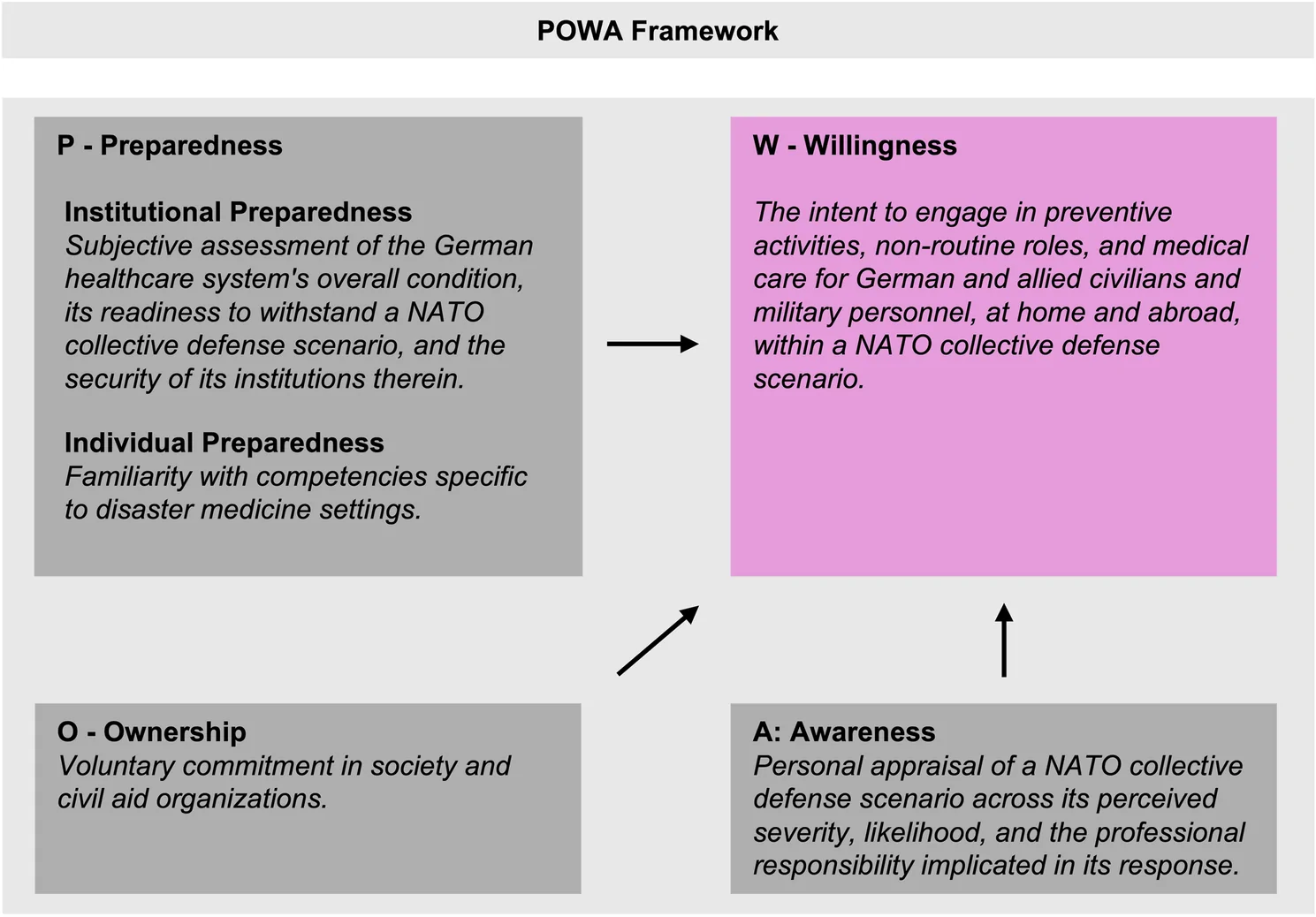
Conceptual model of the POWA framework

Building on these findings, consultations with public health and healthcare system experts, and iterative discussions within our working group, we hypothesized that willingness in a crisis scenario is shaped by three overarching determinants - individual and institutional preparedness, a sense of social responsibility, and awareness of potential threats - rather than by isolated demographic characteristics or static work attitudes. We conceptualized this approach as the POWA framework (P = Preparedness, O = Ownership, W = Willingness, A = Awareness). To describe willingness in a crisis scenario, the framework adapts the threat-and-coping logic of established behavioral models through the dimensions of preparedness and awareness and extends it with an ownership dimension captured by social and voluntary engagement (Fig. 1).

Within this framework, willingness is understood as an individual’s readiness to contribute proactively to the healthcare system beyond the standard requirements of their position. This includes either continuing to meet the needs of normal times with presumably fewer resources, or, if needed, adapting their work focus in response to crisis-related demands and engaging in a crisis setting, whether in hospitals, emergency care structures, or in cooperation with civilian and military organizations. Individual preparedness is defined as the self-assessed ability of healthcare professionals to act competently in crisis situations. Institutional preparedness refers to the extent to which participants perceive the domestic healthcare system as capable of responding effectively to health crises while maintaining its core operational functions. Ownership is conceptualized as a sense of social responsibility, captured through prior voluntary engagement. Awareness is defined as the perceived likelihood of a crisis scenario, including an understanding of its dynamics and broader consequences.

The definitions of the four constructs of the framework were newly developed by the study group for this specific study context. The process was informed by the presented existing foundations [10, 28–32].

In this study, the POWA framework was applied to investigate the willingness of medical, nursing, and paramedical students in Germany in the context of a NATO Article 5 scenario.

To enable the analysis of the relationship of preparedness, ownership, and awareness with overall willingness in this specific context, we developed a questionnaire to operationalize these abstract constructs through individual survey items.

Through this study and the chosen approach, we aim to contribute to a deeper understanding of the willingness of young health care professionals in a military scenario, as well as the measures that may be relevant to prepare healthcare systems for military crisis.

## Methods

### Study design and setting

This cross-sectional study was conducted among medical, nursing, and paramedic students in Germany using an online survey on the platform SoSciSurvey between May 20, 2025, and July 15, 2025. Data collection took place in the context of ongoing public and policy discussions on civil preparedness and collective defense.

### Survey development

As this represents, to our knowledge, the first questionnaire in this field was newly designed. Based on the POWA framework, it is informed by a review of the relevant literature and previously published surveys. Voluntary engagement was quantified using items of the German Volunteer Survey [34]. Participants were asked to indicate which political party they generally support. Questions addressing individual preparedness were based on the curriculum for disaster medicine at German universities [23]. The draft questionnaire was piloted in a sample representative of the target population to assess clarity and face validity. Minor revisions were implemented following the pilot. The full questionnaire is provided in the Supplementary Material (Supplementary Material 3).

### Measures and Variables

#### Primary outcome

Willingness was assessed using a total of 27 decision items grouped into three main domains: (a) willingness to engage in preventive activities in the absence of a NATO collective defense scenario; (b) willingness to work in different roles and under conditions exceeding routine practice in the event of a NATO collective defense scenario; and (c) willingness to provide medical care to German, allied, and enemy civilians and military personnel, both domestically and abroad.

Items assessing willingness for preventive engagement were answered dichotomously (yes/no), reflecting that decisions regarding this could be made at the time of the survey. All remaining items were rated using a three-category response scale (yes/not sure/no) to account for uncertainty regarding future, context-dependent scenarios. A summative willingness score ranging from 0 to 54 points was calculated, with responses coded as 2 points for agreement, 1 point for undecided, and 0 points for disagreement. Negatively worded items were reverse coded accordingly, so that higher scores consistently reflected greater willingness. All items were weighed equally. In addition, the primary motives underlying participants’ willingness were assessed by selecting their primary reason for their willingness from a predetermined list of options.

#### Predictor variables

##### Awareness

Participants’ awareness was conceptualized across three dimensions: (a) perceived fear of a NATO collective defense scenario, (b) perceived likelihood of such a scenario occurring, and (c) perceived responsibility of healthcare professionals to provide support in such a scenario. Each dimension was assessed using a four-point Likert scale. Response options were coded from 1 (strong disagreement) to 4 (strong agreement), with higher scores indicating greater perceived fear, likelihood, and responsibility. Given the complementary nature of these dimensions, item values were multiplied to construct an awareness index, ranging from 1 to 64. To account for the non-linear growth of the multiplicative index and to ease interpretation in the regression analyses, the logarithm of the index was used.

##### Ownership

Ownership was conceptualized and assessed across two dimensions: (a) personal engagement in any type of voluntary work during studies or apprenticeship in general and (b) active personal engagement specifically in voluntary activities in civil aid organizations (volunteer fire brigades or the Federal Agency for Technical Relief (THW)) or a humanitarian aid organizations (e.g. German Red Cross (DRK), Workers’ Samaritan Federation (ASB), St. John Accident Assistance (JUH), German Life Saving Society (DLRG) or Malteser Hilfsdienst (MHD)). These two dimensions were treated as separate items.

##### Preparedness

Preparedness was operationalized at both the individual and institutional levels.

##### Individual preparedness

Individual preparedness was assessed using participants’ self-reported familiarity with selected disaster medicine–specific competencies. These competencies were based on the German curriculum for disaster medicine [23] and adapted to ensure applicability to medical, nursing, and paramedic trainees. Familiarity was rated on a four-point scale ranging from 0 (no prior engagement) to 3 (able to apply independently). A summative individual preparedness score ranging from 0 to 12 was calculated based on the four items, with higher scores indicating greater perceived individual preparedness.

##### Institutional preparedness

Institutional preparedness was assessed across three dimensions: (a) participants perceived overall condition of the German healthcare system; (b) perceived preparedness of the German healthcare system to manage additional strain arising from armed conflict scenarios; and (c) perceived security of hospitals and other healthcare institutions in such scenarios. Each dimension was measured using a four-point Likert scale. Responses were coded from 0 (poor condition/ preparedness/ security) to 3 (good condition/ preparedness/ security), with higher scores indicating a more favorable assessment of the healthcare system’s condition, preparedness, and security in the context of a NATO collective defense scenario. A summative institutional preparedness score (0–9 points) was calculated.

#### Covariates

The following demographic characteristics were collected: age, gender, German citizenship or permanent residence permit, field and stage of study or vocational training, prior military training or education undertaken within the German Armed Forces, completion of vocational training prior to university enrollment, and self-reported political orientation (party preference).

### Study population and recruitment

All medical, nursing, and paramedic students enrolled at German universities or vocational education and training (VET) schools at the time of data collection were eligible to participate if they were over 18 years old and held German citizenship or a permanent residence permit in Germany. Individuals enrolled in other health-related study programs or apprenticeships closely aligned with these study fields were included if their future work comprises direct patient contact, such as nursing assistants in long-term care facilities.

All German medical faculties, student councils, nursing and paramedic VET schools with publicly available online contact information were systematically identified, contacted, and invited to disseminate the study invitation with a link to the openly accessible survey to their students. Approximately 1,500 institutions were contacted, potentially reaching out to several thousand eligible participants. Reminder emails were sent to all institutions on June 3, 2025, and on July 3, 2025. Additionally, the study was promoted via social media platforms (Instagram, WhatsApp).

### Statistical analysis

Descriptive statistics were used to describe the study population. Categorical variables were reported as counts and proportions, and continuous variables as means with standard deviations, ranges, and medians. The distribution of the variables was assessed using descriptive summaries and visual inspection. Participants who did not meet the above-mentioned predefined inclusion criteria or who had implausibly short response times were excluded. Internal consistency of the summative willingness score was assessed using Cronbach’s α (Supplementary Table 1).

Associations between each predictor and willingness were examined using multivariable linear regression models, adjusting for age, gender, field of study or apprenticeship, and training/ apprenticeship with the German Armed Forces as potential confounders (basic model). Political orientation was included in all fully adjusted models. Respondents who refused to report political orientation were retained as a separate category to minimize selection bias. Model-specific confounders were included based on theoretical considerations (see Supplementary Table 2 for detailed adjustment sets per model). Model coefficients, confidence intervals, and p-values were reported. Assumptions of linear regression (linearity, homoscedasticity, and normality of residuals) were evaluated using diagnostic plots and were adequately met.

All analyses were conducted with a two-sided significance level of 0.05, using STATA (versions 18.5 and 19). Graphics were designed with GraphPadPrism (Version 10.3.0) and Microsoft Office.

### Ethical approval

This study was conducted in accordance with the Declaration of Helsinki. The ethics committee of the Berlin State Medical Association reviewed the study protocol and determined that formal ethical approval was not required (reference number: Eth-KB-25-005). All study participants voluntarily gave their consent to take part in the study after being informed of its objectives, procedures, benefits, and risks. No financial or other compensation was provided.

### Declaration of AI use

Artificial intelligence tools (ChatGPT 5.0, DeepL) were used to support language translation between German and English, to assist with grammar and vocabulary editing, and to identify and correct coding errors in STATA. Claude was used to simulate editorial feedback to make further corrections to the manuscript.

## Results

### Characterization of the study cohort

A total of 1005 people initiated the survey, of whom 579 completed the full questionnaire. Of these, 65 (11.2%) were excluded due to ineligible fields of study or vocational education (*n* = 17, 2.9%), implausibly short completion time (*n* = 23, 4.0%), terminated education (*n* = 19, 3.3%), or their gender groups not being large enough for analysis (*n* = 6, 1.0%). This resulted in a final study population of 514 participants.

The median age of participants was 24.6 years (range, 18–38) with slightly more female (*n* = 293, 58.8%) than male participants. Most participants were medical students (*n* = 302, 58.9%), followed by paramedical trainees (*n* = 102, 19.8%) and nursing trainees (*n* = 74, 14.4%). Regarding educational attainment, university students reported a median of 8 semesters (range: 1–18 semesters) and those in vocational training a median of 2 years (range: 1–4 years). A total of 35 participants (6.8%) pursued their education through the German Armed Forces. The overall sociodemographic characteristics of our study cohort are depicted in Tables 1 and 2. The full item-by-item survey results are provided in Supplementary Table 3.

**Table 1:** Sociodemographic characteristics of the study cohort — discrete variables [*n* = 514]

Variable		*n*	%
Sex	Male	221	43.0%
	Female	293	57.0%
Field of study	Medicine	302	58.8%
	Nursing Studies	18	3.5%
	Nursing apprenticeship	74	14.4%
	Paramedic apprenticeship	102	19.8%
	Other field of study with contact to patients	10	2.0%
	Other vocational education with contact to patients	8	1.6%
Apprenticeship/Studies through German Armed Forces	Yes	35	6.8%
	No	479	93.2%
Participation in a summer school on the topic	Yes	71	13.8%
	No	443	86.19
General social engagement [in the previous 3 years]	Yes	304	59.1%
	No	210	40.9%
Engagement in aid organizations^†^ [in the previous 3 years]	Yes (at least one)	142	27.6%
	No	372	72.4%
Political orientation^‡^	CDU/CSU	48	9.3%
	SPD	41	8.0%
	Bündnis 90 / Die Grünen (Green Party)	178	34.6%
	Die Linke (Left Party)	95	18.5%
	FDP	11	2.1%
	AFD	23	4.5%
	BSW	10	1.9%
	Other	15	2.9%
	Refused answer	93	18.1%

† Considered aid organizations: ASB, Workers’ Samaritan Federation; DLRG, German Life Saving Society; DRK, German Red Cross; JUH, St. John Accident Assistance; MHD, Malteser Relief Service; THW, Federal Agency for Technical Relief; Volunteer fire departments

‡ *Abbreviations*: *AFD* Alternative for Germany, *BSW* Sahra Wagenknecht Alliance, *CDU* Christian Democratic Union, *CSU* Christian Social Union, *FDP* Free Democratic Party, *SPD* Social Democratic Party of Germany

**Table 2:** Sociodemographic characteristics of the study cohort — continuous variables [*n* = 514]

	Mean	Standard Deviation	Median	Range
Age [years]	24.6	4.9	24	18–38
Study progress [semesters]	7.4	3.7	8	1–18
Apprenticeship progress [years]	2.0	0.9	2	1–4

### High willingness for preventive activities and domestic medical support despite limited willingness for international relocation

As shown in Table 3, the willingness score had a mean of 36.4 with a standard deviation of 9.9. The distribution of the willingness was approximately normal, with a slight left skew. Internal consistency of the willingness score was adequate (Cronbach’s α = 0.88).

**Table 3:** Characterization of the scores

Score	Mean	Standard Deviation	Median	Interquartile Range	Range
Willingness [0–54]	36.4	9.9	38	30–44	0–54
Individual preparedness [0–12]	3.3	2.6	3	1–5	0–12
Institutional preparedness [0–9]	2.2	1.5	2	1–3	0–6
Awareness [1–64]	18.6	11.8	18	12–24	1–64

**Fig. 2:**
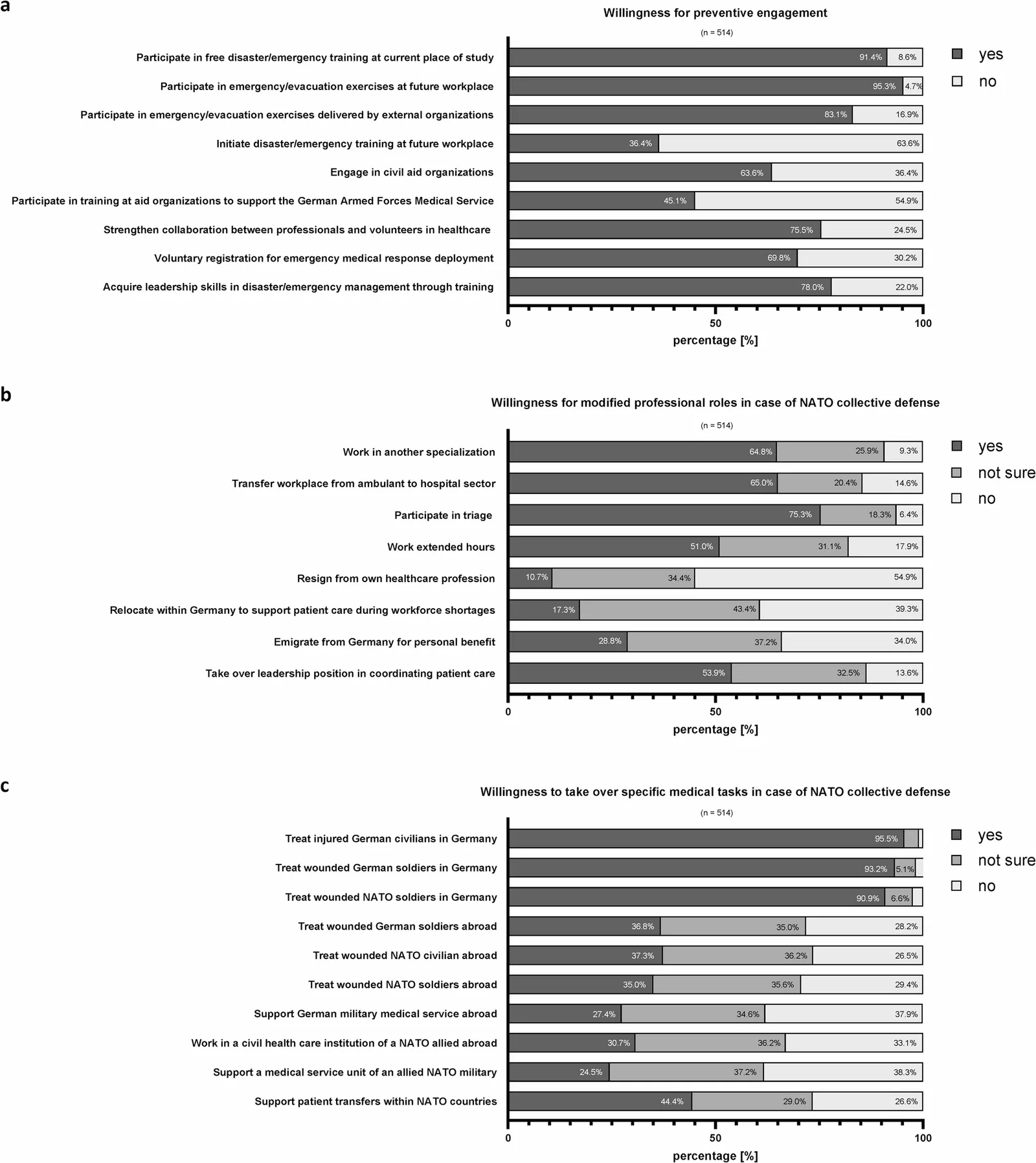
Willingness to engage in preventive engagement and future professional activities and roles in a potential NATO collective defense scenario: Participants were asked to report their willingness to engage in (**a**) preventive activities, **b** modified professional roles, and **c** specific medical tasks in the context of a potential future NATO collective defense scenario. Stacked bar charts present the percentage distribution of responses for each item. Response scales are displayed on the x-axis of the graphs. All data are descriptive data

Item-level analyses revealed high willingness to engage in preparatory measures: for instance, more than 90% (*n* = 470) of participants indicated willingness to enroll in preparation courses if offered at no cost, and more than 80% (*n* = 427) reported willingness to participate in drills. In contrast, willingness was markedly lower for engaging in courses of the German Armed Forces and for organizing preparatory activities independently (Fig. 2A).

Whereas nearly two-thirds of the participants (*n* = 333) would be willing to adapt their professional duties, such as shifting from ambulant to stationary care or working in another specialization, only 17.3% (*n* = 89) would be willing to relocate within Germany (Fig. 2B). Consistent with these findings, over 90% of participants expressed willingness to provide medical care to civilians as well as German or NATO soldiers in Germany (*n* = 491, *n* = 479, *n* = 467, respectively), whereas less than 40% (*n* = 192, *n* = 180, *n* = 141, respectively) would be willing to do the same abroad. While there was a slight preference for treating civilians over military personnel and German soldiers over allied soldiers, these differences were marginal. Regarding potential evasion mechanisms, 10.7% (*n* = 55) of the study population stated that they would consider quitting their job in healthcare, and 28.8% (*n* = 148) would consider emigrating from Germany to a neutral country like Switzerland (Fig. 2C).

**Fig. 3:**
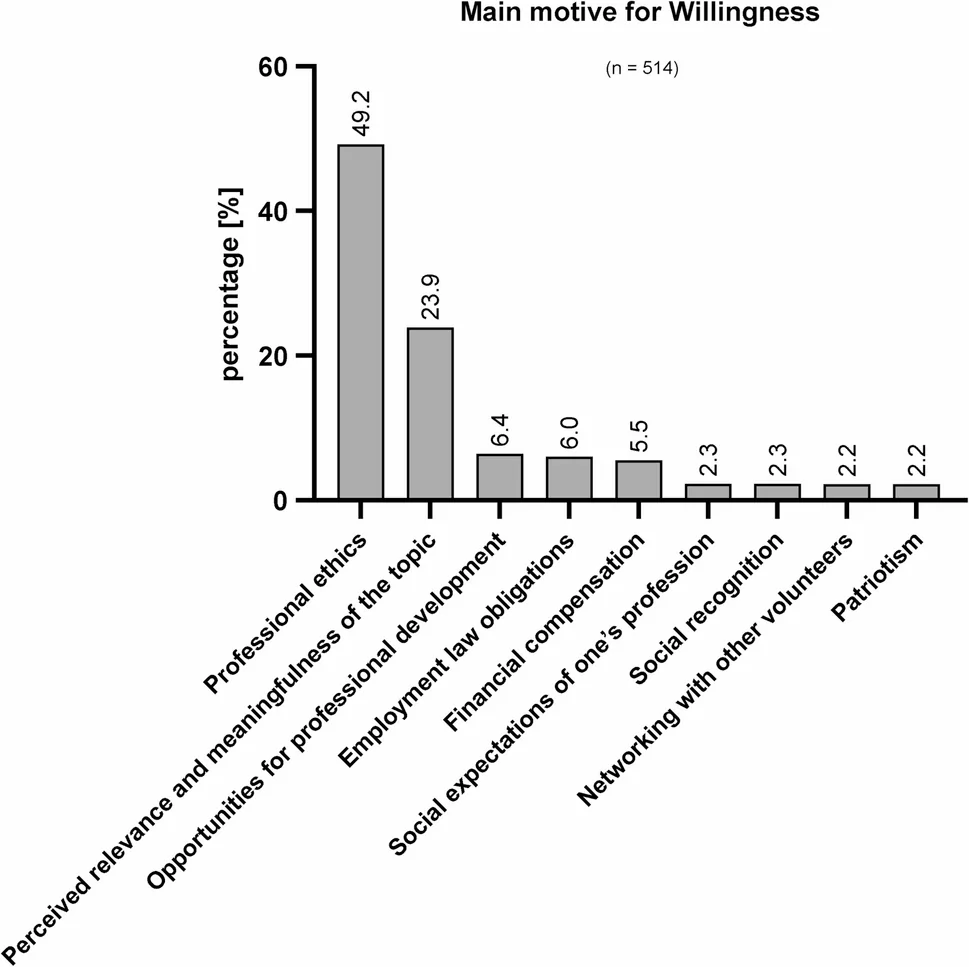
Main motives for Willingness to engage in a NATO collective defense scenario: Participants were asked to select their three main motives underlying their willingness to engage in a potential NATO collective defense scenario. The bar chart displays the percentage distribution of participants’ first-ranked motive. All possible predefined motive categories are shown on the x-axis

When asked about the main motivation for additional engagement in the case of NATO collective defense, professional ethics (*n* = 253, 49.2%) and the perceived relevance of the scenario (*n* = 123, 23.9%) were named by most of the participants (Fig. 3).

### Low perceived institutional preparedness but positive association with willingness

**Fig. 4:**
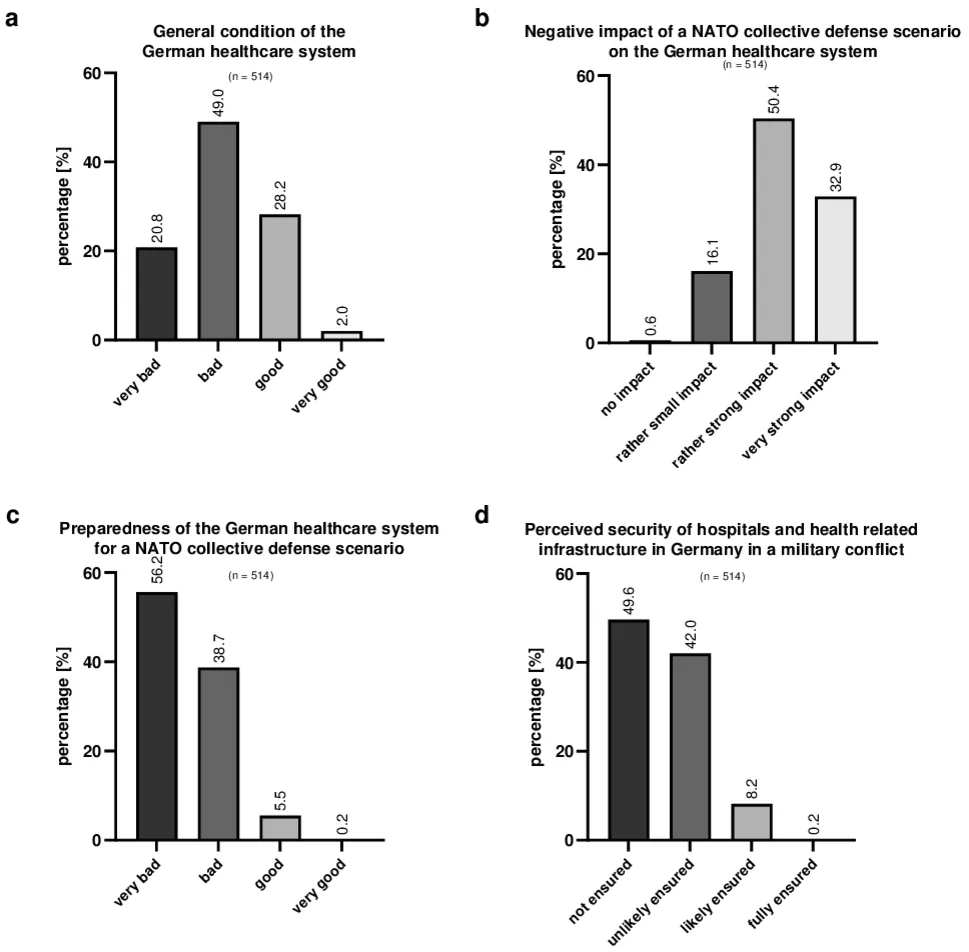
Institutional Preparedness of the German healthcare system regarding a NATO collective defense scenario: Participants were asked to assess (**a**) the current condition of the German healthcare system and (**b**) the expected impact of a potential NATO collective defense scenario on the healthcare system. In the context of such a scenario, participants further evaluated (**c**) the level of preparedness of the healthcare system, and (**d**) the security of healthcare-related infrastructure. Bar charts present the percentage distribution of responses for each item. Response scales are displayed on the x-axis of the graphs. All data are descriptive data

Perceived institutional preparedness of the German healthcare system for armed conflict scenarios was predominantly rated negatively. Overall, 69.9% (*n* = 359) of participants perceived the current state of the system as rather or very defective, whereas only 30.1% (*n* = 155) rated it as at least rather favorable (Fig. 4A). In case of a NATO collective defense scenario, 83.3% (*n* = 428) anticipated negative overall effects on the healthcare system (Fig. 4B). With regards to its preparedness, 94.4% (*n* = 485) considered the German healthcare system to be rather poorly or very poorly prepared regarding a NATO collective defense scenario (Fig. 4C). When asked about the security of hospitals and healthcare institutions in the context of armed conflict, 91.4% (*n* = 471) of respondents rated it as bad or rather bad (Fig. 4D).

Despite these critical assessments, institutional preparedness showed a strong and consistent positive association with willingness to work across all regression models. The effect was highly significant (*p* < 0.01) in both, the partly (β = 1.06) and fully adjusted models (β = 0.80), with coefficients close to 1, indicating a robust positive association between perceived institutional preparedness and willingness (Table 4).

**Table 4:** Association between Institutional preparedness and willingness

	Basic Model*			Fully Adjusted Model**		
	β	95% CI	*p*-value	β	95% CI	*p*-value
Institutional preparedness: (Score range: 0–9)	1.06	(0.50, 1.62)	< 0.001	0.80	(0.21, 1.38)	0.007

β indicates the mean change in willingness (0–54) per one-unit increase in Institutional Preparedness; *n* = 514

* Adjusted for sex, age, field of study/apprenticeship, apprenticeship/studies through German Armed Forces

** Adjusted for sex, age, field of study/apprenticeship, apprenticeship/studies through German Armed Forces, political orientation

Individual preparedness was generally limited, particularly regarding conflict- and catastrophe-specific competencies. While basic principles of disaster medicine were relatively more familiar to the participants (*n* = 118, 23.0% reported being able to apply them; *n* = 146, 28.4% having practiced them), most participants lacked familiarity with more specialized skills. More than half of them had never heard of specific medical skills for catastrophic conditions (*n* = 280, 54.5%), and only 4.5% (*n* = 23) reported being able to apply them. Similar gaps were observed for hospital alarm and evacuation planning (*n* = 354, 68.9% never addressed; *n* = 21, 4.1% able to apply) and mental health support under catastrophic conditions (*n* = 209, 40.7% never addressed; *n* = 23, 4.5% able to apply). Only 13.8% (*n* = 71) of participants had already attended a summer school on disaster medicine.

Even though the coefficients suggest a positive relationship between individual preparedness and willingness, it is neither significant in the basic (β = 0.31; *p* = 0.077) nor the fully adjusted model (β = 0.09; *p* = 0.622) (Table 5).

**Table 5:** Association between individual preparedness and willingness

	Basic Model*			Fully Adjusted Model**		
	β	95% CI	*p*-value	β	95% CI	*p*-value
Individual preparedness: (Score range: 0–12)	0.31	(-0.03, 0.66)	0.077	0.09	(-0.26, 0.44)	0.622

β indicates the mean change in willingness (0–54) per one-unit increase in Individual Preparedness; *n* = 514

* Adjusted for sex, age, field of study/apprenticeship, apprenticeship/studies through German Armed Forces

** Adjusted for sex, age, field of study/apprenticeship, apprenticeship/studies through German Armed Forces, political orientation, participation in a summer school on the topic

### Higher awareness is strongly associated with willingness

**Fig. 5:**
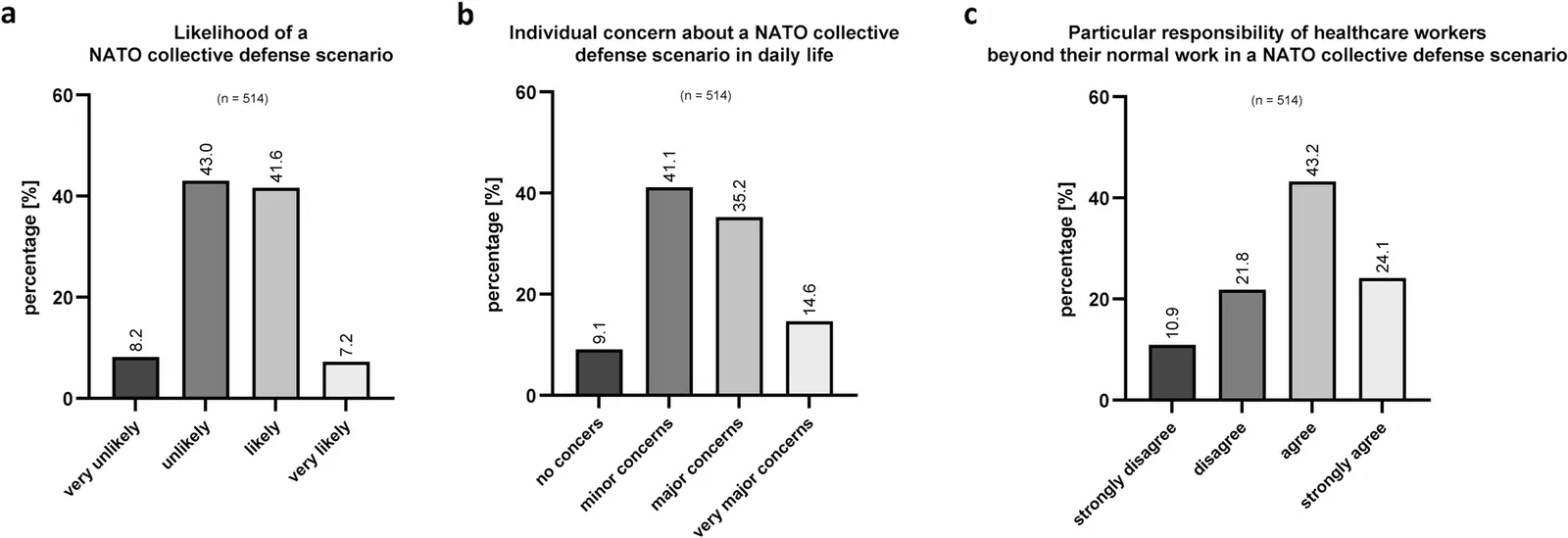
Awareness of a potential NATO collective defense scenario: Participants were asked to report (**a**) the perceived likelihood of a NATO collective defense scenario, (**b**) their personal level of concern regarding such a scenario, and (**c**) their level of agreement with the statement that healthcare workers have an exceptional responsibility in this context. Response scales are displayed on the x-axis of the graphs. All data are descriptive data

In our study, awareness refers to the individual perception of the likelihood and fear regarding a NATO collective defense scenario as well as the responsibility of the medical workforce in such an event. When asked about the perceived probability of such a scenario, 48.8% (*n* = 251) of participants indicated that it is very likely or rather likely, whereas 51.2% (*n* = 263) assessed it as rather unlikely or very unlikely (Fig. 5A). In terms of the emotional appraisal of such a scenario, a similar tendency was found (*n* = 258, 50.2% very or rather afraid of a NATO collective defense; *n* = 256, 49.8% not or little afraid) (Fig. 5B). Awareness further involves participants’ perceptions of their own roles and responsibilities in such a scenario. Most participants (*n* = 346, 67.4%) indicate they would see a very or rather special responsibility of healthcare workers in the context of a NATO collective defense scenario (Fig. 5C).

In the regression analysis, our results show a strong and statistically significant positive association between awareness and willingness. Each unit increase in log-transformed awareness was associated with a 4.13-point increase (*p* < 0.001) in willingness in the basic model and a 3.88-point increase (*p* < 0.001) in the fully adjusted model (Table 6).

**Table 6:** Association between awareness and willingness

	Basic Model*			Fully Adjusted Model**		
	β	95% CI	*p*-value	β	95% CI	*p*-value
Awareness: (log-transformed)	4.13	(3.05–5.21)	< 0.001	3.88	(2.67–5.10)	< 0.001

β represents the change in willingness (0–54) per unit increase in log-transformed Awareness; *n* = 514

* Adjusted for sex, age, field of study/apprenticeship, apprenticeship/studies through German Armed Forces

** Adjusted for sex, age, field of study/apprenticeship, apprenticeship/studies through German Armed Forces, political orientation

### Voluntary social engagement is associated with higher willingness

Regarding ownership, most participants reported voluntary activities during their studies or apprenticeship within the preceding three years (*n* = 304, 59.1%), with an average commitment of 1.82 h per week (range, 0–21) over this period. The multivariable regression analysis reveals that socially engaged participants have a significantly higher willingness (basic model: β = 3.65, *p* < 0.001; fully adjusted model β = 3.09, *p* = 0.002) (Table 7). Overall, 27.6% of participants (*n* = 142) reported volunteering in civil or humanitarian aid organizations. More than three-quarters (*n* = 111) reported that their engagement had prepared them for working in the healthcare system during a NATO collective defense scenario.

Our results indicate that participants volunteering in civil aid organizations tended to show higher willingness than those engaged in other areas (β = 1.85; *p* = 0.122), although this association was not significant (Table 7).

**Table 7:** Association between general social engagement (ownership) and willingness

	Basic Model*			Fully Adjusted Model**		
	β	95% CI	*p*-value	β	95% CI	*p*-value
Social engagement: (Yes/ No)	3.65	(1.87–5.44)	< 0.001	3.09	(1.12–5.05)	0.002
Engagement in civil aid organizations: (Yes/ No)	-	-	-	1.85	(-0.49–4.20)	0.122

β indicates the mean change in willingness (0–54) depending on Social Engagement; n = 514

* Adjusted for sex, age, field of study/apprenticeship, apprenticeship/studies through German Armed Forces

** Adjusted for sex, age, field of study/apprenticeship, apprenticeship/studies through German Armed Forces, political orientation, participation in a summer school on the topic

## Discussion

This is the first study to investigate factors associated with the willingness of future healthcare professionals to actively contribute to the German healthcare system in a potential NATO collective defense scenario. Overall, participants demonstrated high motivation to participate in preparation courses and activities; however, there was low willingness for geographical mobility. Professional ethics and the perceived relevance of the scenario were the primary motives for contributing in a military crisis scenario.

Our study identifies perceived institutional preparedness of the German healthcare system, ownership, and the awareness of a NATO collective defense scenario as the main factors associated with willingness to work beyond the normal workload.

### Framework

Our findings are in line with the logic of established behavioral frameworks, such as the Protection Motivation Theory [30] and the Health Belief Model [31], which argue that willingness depends both on how people perceive a threat (awareness) and on how confident they feel that it can be managed (preparedness). In our data, awareness reflects the perception of the threat, while perceived institutional and individual preparedness reflect confidence that the situation can be handled, either by the healthcare system as a whole or by oneself. Two findings, however, show that the expansion of these theories through the POWA framework might be reasonable. First, the two forms of preparedness seem to behave differently: confidence in the system was associated with willingness, whereas confidence in one’s own preparation was not. Second, ownership, measured as prior social engagement, was strongly associated with willingness even though it falls outside these models, which supports treating it as a separate dimension and points to the role of social and prosocial motivation. Hence, these points reinforce the rationale for our exploratory POWA framework, which extends this logic to the institutional and social dimensions relevant to such a scenario.

### Willingness

As participants indicated willingness to engage in more than half of the 27 proposed activities beyond regular employment, or considered such engagement possible, the results demonstrate a considerable overall level of willingness. More than half of the participants expressed openness to changes to their professional life, such as transitioning from ambulatory to inpatient care, to support the system. This finding is consistent with levels of willingness reported in general disaster scenarios in the literature [35].

In our survey, willingness was dependent on contextual factors. For example, participants preferred to provide help within Germany rather than on the territory of an allied nation. This likely reflects concerns regarding personal safety and the proximity to and protection of family members, factors that have been consistently highlighted in previous studies [32, 35–37]. Furthermore, participants expressed a clear preference for emergency care or preparatory courses without military affiliation, which underscores the importance of civilian aid organizations in the context of a NATO collective defense scenario.

The primary motivations underlying participants’ willingness to work were professional ethics and perceived relevance of the scenario, which is consistent with existing literature [29, 38]. This highlights the strong influence of professional identity, which may be fostered through structured educational curricula and targeted training during medical education [38].

### Preparedness

In this study, institutional preparedness is defined as the perceived condition, security situation, and state of preparation of the German healthcare system for a NATO collective defense scenario. Participants predominantly rated the institutional preparedness of the German healthcare system as poor. When compared to other assessments of the institutional preparedness, the literature is heterogeneous. In the 2025 State Party Self-Assessment, the German healthcare system performed above average compared with other European countries, identifying risk communication and community engagement as major challenges [39]. Several surveys of healthcare personnel, however, revealed deficits in crisis scenarios. For instance, there are no standardized plans to call off-duty personnel [40], a lack of skilled personnel to treat penetrating injuries [41], and no structural protective measures for hospitals [42]. In addition, while preparedness plans might be present, the practical implementation is insufficient [42].

At the same time, higher perceived institutional preparedness was significantly linked to greater willingness among future healthcare professionals to extend their workload in a NATO collective defense scenario. This finding aligns with prior literature. For instance, an Israeli study reported lower absenteeism among health care workers during wartime when the healthcare system was perceived as well prepared, whereas feeling unsafe in the work environment was associated with increased absenteeism [37]. In accordance with these findings, a study has also shown that improved institutional preparedness enhances perceived safety among healthcare workers [32].

Given these findings and assuming causality, the perceived lack of security of hospitals and healthcare institutions may be a relevant area for attention. Strengthening institutional security may be essential to sustain, and not undermine, the high levels of willingness observed among future healthcare professionals.

Regarding individual preparedness, the low levels reported by participants are consistent with previous studies [36, 43]. Individual preparedness has been identified as an important factor influencing the willingness to engage in disaster response [33]. For example, South Korean emergency nurses with disaster training had higher perceived preparedness and a greater willingness to respond [44]. Similarly, Al-Hunaishi et al. showed that self-efficacy, a core component of individual preparedness, was significantly associated with willingness to participate in disaster response among Yemeni nurses and doctors [45]. Another study reported a positive association between education and willingness to work, while also suggesting that excessive focus on threatening aspects may reduce willingness if risks are emphasized too intensively [36]. Previous research from Germany has shown that, in addition to fundamental knowledge and skills such as definitions, roles, and role conflicts, relevant topics for such training should include managing emotions and motivation, and adaptable skills [46, 47]. Training involving clear instructions and disaster simulations is considered useful for this purpose [46].

The results of this study do not fully mirror the patterns found in prior literature, as the coefficient for individual preparedness is positive but not significant. This contrast can be interpreted in terms of the coping appraisal described in the behavioral frameworks above [31]: the perception that the system as a whole is capable (response efficacy) may be more closely linked to willingness than confidence in one’s own preparation (self-efficacy). This is plausible given that institutional capacity, rather than individual competence, is likely to be the main limitation when demand is expected to exceed the resources of any single professional [48, 49]. The positive but non-significant coefficient for individual preparedness is also consistent with a floor effect, as reported individual preparedness was generally low.

### Awareness

Awareness comprises the perceived likelihood and threat of a NATO collective defense scenario, as well as the perceived relevance of healthcare professionals’ roles in such a context. While respondents were divided in their assessment of both the likelihood of such a scenario as well as the perceived threat it would pose, two-thirds of participants perceived a special professional responsibility associated with their role as healthcare workers in a NATO collective defense scenario. Participants with higher levels of awareness exhibited greater willingness to actively contribute to increased work demands. Comparable results were reported by Ganz et al. [50], who found that willingness to work increased with higher perceived threat across different disaster scenarios. In contrast, several studies reported negative or non-significant associations between perceived threat and willingness, particularly in war or high-risk contexts where fear, insecurity, and concerns about personal or family safety reduced willingness to report to work [36, 37, 51]. Notably, these effects were mitigated when institutional preparedness, role clarity, and perceived safety were present [37, 52, 53].

These divergent findings might be explained by differences in how awareness and threat are defined. While previous studies often focused on perceived fear in concrete disaster situations, the awareness construct in this present study reflects a broader perception of likelihood and threat, independent of the individuals’ immediate roles as healthcare workers, and additionally incorporates perceived professional responsibility.

This interpretation is supported by participants’ reported motivations for working beyond the normal workload, which were mainly professional ethics and the perceived relevance of the scenario. Taken together, these findings indicate that all components of awareness - likelihood, threat, and professional relevance - are positively linked to willingness to work beyond normal capacity.

### Ownership

In this study, ownership was primarily operationalized as social engagement. Social engagement, in the form of voluntary work, was positively associated with willingness to actively contribute to increased work demands in a NATO collective defense scenario. This finding aligns with prior research demonstrating a positive, bidirectional relationship between volunteering and social cohesion [54–56]. Social cohesion is commonly defined as the willingness to help others (including across social groups), a sense of belonging and identity, and social and political participation [57]. Voluntary work may therefore be associated with higher individuals’ willingness to work in such scenarios by strengthening social cohesion.

Davies et al. showed that volunteering is associated with increased trust in governmental institutions [54]. Increased institutional trust may, in turn, translate into higher perceived institutional preparedness, which our results identified as a key factor associated with higher willingness to work beyond normal capacity.

Moreover, voluntary engagement in relevant fields may foster practical experience and improve individual preparedness. This interpretation is supported by data from our study, in which 83% of respondents who were engaged in civil aid organizations reported that their voluntary work prepared them for working in the healthcare system during a NATO collective defense scenario. In corresponding previous research, it has been shown that voluntary work, particularly among young people, facilitates gaining both hard and soft skills [58].

Overall, these findings suggest that social engagement - and the associated sense of ownership - represents an important predictor of willingness to actively contribute to increased healthcare work demands in a NATO collective defense scenario.

### Strengths and limitations

Although nearly all universities, nursing and paramedical schools across Germany were systematically contacted, the open online recruitment strategy did not allow the response rate to be determined. Therefore, the study findings may be affected by selection bias. It cannot be ruled out that institutions with a stronger focus on disaster medicine in their curricula were more likely to distribute our survey, resulting in recruitment bias. In addition, participants with a higher level of willingness may have completed our questionnaire at higher rates compared to those with lower willingness, potentially introducing self-selection bias. Most of the dropouts occurred within the first few questions, without a clear concentration on a specific item. This suggests that neither the length nor the content of the questionnaire triggered the dropout. Nonetheless, the study achieved nationwide recruitment across a broad range of healthcare professions and reflects the underlying population reasonably well with respect to gender and age within the different professional groups [59–61]. The study population contains a high share of medical students (*n* = 302, 58.9%), and within the medical student subgroup an overproportionate share of students affiliated with the German Armed Forces. While participants represented a range of political preferences, the sample included a comparatively high proportion of politically progressive individuals. Given the potential recruitment and self-selection bias and because more civically and politically engaged individuals may also be more inclined to contribute during a crisis, descriptive estimates such as overall willingness scores and rates of voluntary engagement may be higher than would be expected in the full population. To reduce the influence of these differences on the reported associations, all multivariable models were adjusted for political orientation, field of study, and German Armed Forces affiliation as confounders.

This study is, to our knowledge, the first to investigate willingness to support the healthcare system in a potential military conflict scenario among future healthcare professionals in Germany. As these individuals are expected to enter the workforce around 2030 and form a substantial part of the future healthcare workforce, the findings provide important initial evidence on a highly relevant and understudied population. While the results should not be interpreted as population-representative estimates, they offer valuable insights into factors associated with willingness in this target group. Given the lack of prior literature in this field, we developed the POWA framework, which is theoretically grounded in established behavioral models and was designed to provide a structured basis for investigating willingness in military conflict scenarios. Although the framework requires future psychometric validation, it may serve as a useful starting point for hypothesis generation and subsequent research.

Furthermore, the present cross-sectional design allows the identification of associations but cannot establish causality, validate the framework itself, or determine the direction of relationships between variables. The observed associations should therefore be interpreted with caution, as bidirectional relationships cannot be ruled out. For example, individuals who are more willing to support the German healthcare system may also be more likely to join a civic aid organization to gain relevant experience. Conversely, engagement in such organizations may later shape attitudes towards willingness during healthcare training.

Public and political discussions surrounding a potential NATO collective defense scenario are ongoing. Therefore, the findings reflect the specific geopolitical and societal context of summer 2025. Importantly, peacetime should not be considered a static context. Perceptions of security threats and willingness to engage may change across different periods of peacetime in response to evolving geopolitical developments, international relations, and political leadership, and may differ further under conditions of an actual NATO collective defense scenario. Nevertheless, preparedness planning before potential crises is a core element of health system resilience [14]. Against this background, the present study provides timely evidence on a topic of increasing relevance and highlights factors that may influence future preparedness planning, particularly regarding the next generation of healthcare professionals.

## Conclusions

The aim of our study was to investigate the willingness of future healthcare professionals to engage beyond their regular workload in case of a NATO collective defense scenario and to identify aspects that are associated with this willingness. Building on the POWA framework, our findings demonstrate a significant association between willingness and voluntary social engagement, the operational measure of ownership. Considering declining levels of voluntary engagement in society [46], strengthening voluntary work, especially within aid organizations, may be a promising strategy, though its causal relation to willingness remains to be established.

Our findings suggest that, presuming a causal relation, perceptions of institutional preparedness of the healthcare system might play a relevant role in shaping willingness to engage beyond normal working capacities and duties. While respondents generally rated the current level of preparedness poorly, more positive perceptions were associated with greater willingness. In consequence, institutional preparedness may be a candidate target for policy interventions, particularly those aimed at enhancing hospital security. In this regard, policymakers might consider directing investments for system resilience not exclusively toward military defense but putting a stronger emphasis on integrated resilience across sectors, including healthcare system resilience. Although we could not objectively assess the actual participants’ preparedness, self-reported individual preparedness was generally low. However, in contrast to institutional preparedness, it appeared to have less influence on the respondents’ willingness in our study.

In addition, our analysis underscores the importance of awareness - particularly considering the named key motivating factors of professional ethics and perceived relevance of the scenario - in relation to willingness. Assuming causality, these findings suggest that cultivating a sense of collective responsibility and strengthening awareness among both healthcare professionals and the wider society could contribute to maintain healthcare system functionality and resilience in times of crises.

Future research might examine the willingness of other population groups, including currently employed healthcare professionals and non-medical staff. Such insights could further inform targeted preparedness strategies and policy interventions aimed at strengthening overall health system resilience.

## Supplementary Information

Supplementary Material 1. Cronbach's Alpha.

Supplementary Material 2. Full Regression Tables.

Supplementary Material 3. Full Questionnaire.

Supplementary Material 4. Descriptive Survey Results.

## Data Availability

The datasets used and analyzed during the current study are available from the corresponding author on reasonable request.

## Abbreviations

NATO: North Atlantic Treaty Organization
HNS: Host Nation Support
OECD: Organisation for Economic Co-operation and Development
DRK: German Red Cross (Deutsches Rotes Kreuz)
JUH: St John Accident Assistance (Johanniter-Unfall-Hilfe)
MHD: Malteser Hilfsdienst
ASB: Workers’ Samaritan Federation (Arbeiter-Samariter-Bund)
DLRG: German Life Saving Association (Deutsche Lebens-Rettungs-Gesellschaft)
THW: Federal Agency for Technical relief (Technisches Hilfswerk)
COVID-19: Coronavirus disease 2019
