# Supplementary material for: Preparedness, awareness and ownership of young future healthcare professionals are associated with their willingness to contribute to health system resilience in a NATO collective defense scenario: a cross-sectional study among medical, nursing and paramedic students in Germany
Source: BMC Public Health. 2026 Oct 2;26:2758. doi: 10.1186/s12889-026-29675-1 (PMC13632156; doi:10.1186/s12889-026-29675-1)
Supplement: Supplementary file 1 [file 12889_2026_29675_MOESM1_ESM.pdf]

Item Description	Item-test correlation	Item-rest correlation	Average interitem covariance	Alpha
**BE09 (Q32) — Preventive Measures**				
Attending free disaster/crisis medicine events at current training institution	0,3942	0,3456	0,1245	0,8798
Participating in exercises for emergency/evacuation plans at future workplace	0,3196	0,2811	0,1267	0,8810
Participating in exercises organized by external providers	0,4485	0,3854	0,1217	0,8791
Independently initiating continuing education in crisis/disaster medicine at future workplace	0,4323	0,3492	0,1204	0,8810
Becoming involved with a national aid organization (DRK, ASB, JHU, DLRG, MHD)	0,3970	0,3113	0,1214	0,8822
Obtaining further training within voluntary role supporting Bundeswehr Medical Service	0,5903	0,5194	0,1153	0,8758
Advocating for strengthening cooperation between professional and voluntary actors	0,4203	0,3456	0,1215	0,8805
Voluntarily registering in a crisis registry for medical personnel	0,5601	0,4920	0,1171	0,8764
Acquiring leadership skills in crisis/disaster medicine through further training	0,4976	0,4311	0,1197	0,8780
**BE10 (Q33) — Structural Scenarios**				
Working outside own specialization to maintain routine care	0,3968	0,3389	0,1236	0,8800
Switching from outpatient to inpatient care	0,2981	0,2287	0,1252	0,8827
Performing triage	0,4133	0,3626	0,1239	0,8795
Working beyond normal working hours to physical/psychological limits	0,5796	0,5253	0,1185	0,8756
Leaving profession to avoid additional work burden*	0,2605	0,1954	0,1263	0,8831
Changing place of work within Germany	0,4870	0,4292	0,1211	0,8780
Considering foreign residence*	0,2974	0,2230	0,1250	0,8832
Taking on a leadership role to coordinate patient care	0,4971	0,4400	0,1209	0,8777
**BE13 (Q34) — Military/Medical Responsibility**				
Care of injured German civilians — in Germany	0,3394	0,3144	0,1276	0,8810
Care of wounded German soldiers — in Germany	0,4612	0,4325	0,1256	0,8794
Care of wounded soldiers from NATO allied partners — in Germany	0,4806	0,4483	0,1248	0,8788
Care of wounded German soldiers — abroad	0,7328	0,6920	0,1142	0,8710
Care of injured civilians of a NATO allied partner — abroad	0,6877	0,6425	0,1155	0,8724
Care of wounded soldiers of a NATO allied partner — abroad	0,7472	0,7084	0,1139	0,8706
Support of the Bundeswehr Medical Service — abroad	0,7376	0,6976	0,1141	0,8709
Working in a civilian healthcare facility of a NATO allied partner — abroad	0,6701	0,6225	0,1158	0,8729
Support of the Medical Service of a NATO allied partner — abroad	0,7368	0,6978	0,1145	0,8711
Conducting patient transport within NATO allied countries	0,5437	0,4814	0,1186	0,8767
**Test scale**			**0,1206**	**0,8818**

Observations: 520

* Questions were valued inversely
