# Supplementary material for: Preparedness, awareness and ownership of young future healthcare professionals are associated with their willingness to contribute to health system resilience in a NATO collective defense scenario: a cross-sectional study among medical, nursing and paramedic students in Germany
Source: BMC Public Health. 2026 Oct 2;26:2758. doi: 10.1186/s12889-026-29675-1 (PMC13632156; doi:10.1186/s12889-026-29675-1)
Supplement: Supplementary file 2 [file 12889_2026_29675_MOESM2_ESM.pdf]

Supplementary Material S2: Full Regression Tables

S2.1: Association between General Social Engagement (Ownership) and Willingness

	Basic Model*			Fully Adjusted Model**		
	β	95% CI	p-value	β	95% CI	p-value
**Social Engagement: (Yes/ No)**	3.65	(1.87 - 5.44)	<0.001	2.78	(0.95 - 4.61)	0.003
**Sex**						
Female (Ref.)	Ref.	Ref.	Ref.	Ref.	Ref.	Ref.
Male	1.96	(0.17 - 3.75)	0.032	1.93	(0.27 - 3.59)	0.023
**Age (years)**	-0.01	(-0.19 - 0.18)	0.949	-0.04	(-0.24 - 0.15)	0.672
**Field of study/ apprenticeship**						
Medicine (Ref.)	Ref.	Ref.	Ref.	Ref.	Ref.	Ref.
Nursing studies	0.390	(-4.24 - 5.02)	0.868	1.30	(-3.39 - 5.98)	0.586
Nursing apprenticeship	-1.54	(-4.11 - 1.04)	0.241	1.17	(-1.47 - 3.81)	0.383
Paramedic apprenticeship	0.74	(-1.47- 2.95)	0.509	-0.04	(-2.25 - 2.17)	0.971
Other field of study with contact to patients	2.80	(-3.26 - 8.85)	0.365	0.33	(-3.13 - 3.78)	0.853
Other vocational education with contact to patients	-2.54	(-9.34 - 4.27)	0.464	-1.05	(-9.59 - 7.48)	0.808
**Apprenticeship/ studies through German Armed Forces**	2.95	(1.27 - 4.63)	<0.001	2.69	(1.24 - 4.14)	<0.001
**Political orientation**						
Bündnis 90 / Die Grünen (Green Party) (Ref.)				Ref.	Ref.	Ref.
CDU/CSU				1.19	(-1.56 - 3.94)	0.396
SPD				0.25	(-2.94 - 3.44)	0.876
Die Linke (Left Party)				-3.50	(-5.68 - -1.33)	0.002
FDP				0.77	(-4.41 - 5.94)	0.771
AFD				-5.37	(-10.83 - 0.10)	0.054
BSW				-6.11	(-9.58 - -2.64)	0.001
Other party				2.45	(-3.39 - 8.3)	0.410
Refused answer				-2.20	(-4.61 - 0.20)	0.073
**Participation in a summer school on the topic**				3.30	(1.27 - 5.32)	0.001
**Engagement in aid organizations**				1.91	(-0.22 - 4.04)	0.078
**Institutional Preparedness**				0.94	(0.41 - 1.46)	0.001

**Individual Preparedness**		0.10	(-0.21 - 0.42)	0.525
**Awareness**		3.94	(2.71 - 5.16)	<0.001
**Adjusted R^2**	0.08			0.24

β indicates the mean change in Willingness (0–54); n = 514
The most frequent category was selected as the reference category.
* Adjusted for sex, age, field of study/apprenticeship, apprenticeship/studies through German Armed Forces
** Adjusted for sex, age, field of study/apprenticeship, apprenticeship/studies through German Armed Forces, political orientation, participation in a summer school on the topic, Engagement in aid organizations, Institutional Preparedness, Individual Preparedness, Awareness

S2.2: Association between Individual Preparedness and Willingness

	Basic Model*			Fully Adjusted Model**		
	β	95% CI	p-value	β	95% CI	p-value
**Individual Preparedness:**	0.31	(-0.03 - 0.66)	0.077	0.09	(-0.26 - 0.44)	0.622
**Sex**						
Female (Ref.)	Ref.	Ref.	Ref.	Ref.	Ref.	Ref.
Male	1.97	(0.15 - 3.78)	0.034	1.27	(-0.45 - 3.00)	0.148
**Age (years)**	-0.08	(-0.26 - 0.10)	0.376	-0.08	(-0.29 - 0.14)	0.479
**Field of study/ apprenticeship**						
Medicine (Ref.)	Ref.	Ref.	Ref.	Ref.	Ref.	Ref.
Nursing Studies	-0.35	(-5.02 - 4.32)	0.882	2.49	(-2.47 - 7.45)	0.324
Nursing apprenticeship	-3.04	(-5.59 - 0.49)	0.019	0.90	(-1.91 - 3.71)	0.528
Paramedic apprenticeship	0.29	(-2.06 - 2.64)	0.808	1.11	(-1.26 - 3.48)	0.357
Other field of study with contact to patients	2.25	(-3.93 - 8.42)	0.475	1.69	(-2.50 - 5.86)	0.426
Other vocational education with contact to patients	-2.96	(-9.85 - 3.93)	0.398	-0.20	(-8.51 - 8.11)	0.963
**Apprenticeship/ studies through German Armed Forces**	2.94	(1.23 - 4.64)	0.001	2.83	(1.42 - 4.24)	<0.001
**Political orientation**						
Bündnis 90 / Die Grünen (Green Party) (Ref.)				Ref.	Ref.	Ref.
CDU/CSU				1.61	(-1.22 - 4.45)	0.264
SPD				1.35	(-2.02 - 4.72)	0.432
Die Linke (Left Party)				-4.44	(-6.67 - -2.20)	<0.001
FDP				-0.23	(-6.15 - 5.70)	0.940
AFD				-6.94	(-12.23 - -1.16)	0.019
BSW				-8.81	(-13.86 - -3.77)	0.001
Other party				1.16	(-5.88 - 8.19)	0.747
Refused answer				-3.12	(-5.70 - -0.55)	0.017
**Participation in a summer school on the topic**				3.59	(1.45 - 5.73)	0.001
**Engagement in aid organizations**				1.82	(-0.54 - 4.18)	0.130
**Social Engagement: (Yes/ No)**				3.08	(1.11 - 5.05)	0.002
**Adjusted R^2**			0.05			0.14

β indicates the mean change in Willingness (0–54); n = 514
The most frequent category was selected as the reference category.
* Adjusted for sex, age, field of study/apprenticeship, apprenticeship/studies through German Armed Forces
** Adjusted for sex, age, field of study/apprenticeship, apprenticeship/studies through German Armed Forces, political orientation, participation in a summer school on the topic, Engagement in aid organizations, Social Engagement

S2.3: Association between Institutional Preparedness and Willingness

	Basic Model*			Fully Adjusted Model**		
	β	95% CI	p-value	β	95% CI	p-value
**Institutional Preparedness**	1.06	(0.50 - 1.62)	<0.001	0.80	(0.21 - 1.38)	0.007
**Sex**						
Female (Ref.)	Ref.	Ref.	Ref.	Ref.	Ref.	Ref.
Male	2.10	(0.31 - 3.90)	0.022	1.83	(0.06 - 3.60)	0.043
**Age (years)**	-0.06	(-0.24 - 0.12)	0.511	-0.11	(-0.32 - 0.10)	0.314
**Field of study/ apprenticeship**						
Medicine (Ref.)	Ref.	Ref.	Ref.	Ref.	Ref.	Ref.
Nursing Studies	-0.36	(-4.98 - 4.26)	0.878	0.84	(-4.09 - 5.76)	0.738
Nursing apprenticeship	-2.05	(-4.59 - 0.48)	0.113	-0.25	(-3.11 - 2.61)	0.863
Paramedic apprenticeship	1.68	(-0.57 - 3.92)	0.143	2.47	(0.23 - 4.70)	0.030
Other field of study with contact to patients	2.90	(-3.16 - 8.97)	0.348	3.38	(-0.56 - 7.33)	0.093
Other vocational education with contact to patients	-3.43	(-10.24 - 3.38)	0.323	-1.98	(-9.93 - 5.98)	0.626
**Apprenticeship/ studies through German Armed Forces**	3.21	(1.52 - 4.90)	<0.001	3.13	(1.62 - 4.64)	<0.001
**Political orientation**						
Bündnis 90 / Die Grünen (Green Party) (Ref.)				Ref.	Ref.	Ref.
CDU/CSU				1.25	(-1.50 - 3.99)	0.372
SPD				1.00	(-2.29 - 4.28)	0.551
Die Linke (Left Party)				-3.75	(-6.06 - -1.44)	0.002
FDP				-0.69	(-6.38 - 5.01)	0.813
AFD				-6.20	(-11.88 - -0.51)	0.033
BSW				-7.52	(-12.86 - -2.18)	0.006
Other party				2.13	(-5.02 - 9.29)	0.558
Refused answer				-2.16	(-4.75 - 0.43)	0.102
**Adjusted R²**			0.07			0.10

β indicates the mean change in Willingness (0–54); n = 514
The most frequent category was selected as the reference category.
* Adjusted for sex, age, field of study/apprenticeship, apprenticeship/studies through German Armed Forces
** Adjusted for sex, age, field of study/apprenticeship, apprenticeship/studies through German Armed Forces, political orientation

S2.4: Association between Awareness and Willingness

	Basic Model*			Fully Adjusted Model**		
	**β**	**95% CI**	**p-value**	**β**	**95% CI**	**p-value**
**Awareness (log transformed)**	4.13	(3.05 - 5.21)	<0.001	3.88	(2.67 - 5.10)	<0.001
**Sex**						
Female (Ref.)	Ref.	Ref.	Ref.	Ref.	Ref.	Ref.
Male	2.57	(0.84 - 4.29)	0.004	2.35	(0.64 - 4.10)	0.007
**Age (years)**	-0.04	(-0.21 - 0.14)	0.679	-0.09	(-0.29 - 0.11)	0.362
**Field of study/ apprenticeship**						
Medicine (Ref.)	Ref.	Ref.	Ref.	Ref.	Ref.	Ref.
Nursing Studies	-1.03	(-5.48 - 3.42)	0.650	0.13	(-4.76 - 5.03)	0.957
Nursing apprenticeship	-2.27	(-4.69 - 0.14)	0.065	-0.38	(-3.11 - 2.35)	0.786
Paramedic apprenticeship	-0.54	(-2.71 - 1.62)	0.621	0.48	(-1.67 - 2.64)	0.661
Other field of study with contact to patients	1.66	(4.18 - 7.50)	0.578	2.21	(-1.25 - 5.67)	0.210
Other vocational education with contact to patients	-3.50	(-10.05 - 3.05)	0.294	-1.98	(-10.08 - 6.12)	0.631
**Apprenticeship/ studies through German Armed Forces**	2.77	(1.15 - 4.40)	0.001	2.77	(1.36 - 4.17)	<0.001
**Political orientation**						
Bündnis 90 / Die Grünen (Green Party) (Ref.)				Ref.	Ref.	Ref.
CDU/CSU				0.65	(-2.02 - 3.32)	0.632
SPD				0.26	(-3.02 - 3.53)	0.878
Die Linke (Left Party)				-3.72	(-6.06 - -1.39)	0.002
FDP				-0.19	(-5.94 - 5.57)	0.949
AFD				-6.41	(-11.79 - -1.03)	0.020
BSW				-7.56	(-11.54 - -3.58)	<0.001
Other party				2.69	(-3.05 - 8.42)	0.358
Refused answer				-2.07	(-4.57 - 0.43)	0.104
**Adjusted R^2**			0.14			0.17

β represents the change in Willingness (0–54); n = 514
The most frequent category was selected as the reference category.
* Adjusted for sex, age, field of study/apprenticeship, apprenticeship/studies through German Armed Forces
** Adjusted for sex, age, field of study/apprenticeship, apprenticeship/studies through German Armed Forces, political orientation
