# Supplementary material for: Preparedness, awareness and ownership of young future healthcare professionals are associated with their willingness to contribute to health system resilience in a NATO collective defense scenario: a cross-sectional study among medical, nursing and paramedic students in Germany
Source: BMC Public Health. 2026 Oct 2;26:2758. doi: 10.1186/s12889-026-29675-1 (PMC13632156; doi:10.1186/s12889-026-29675-1)
Supplement: Supplementary file 3 [file 12889_2026_29675_MOESM3_ESM.pdf]

EL

EL01

Consent to Participate and to the Processing of My Data

POWA-Analysis: Resilience of the German Healthcare System in a NATO Collective Defense Scenario – A Survey on Preparedness, Ownership, Willingness and Awareness among Young Future Healthcare Workers in the Medical, Nursing, and Paramedic Fields.

Dear Study Participant,

Against the backdrop of Russia's war of aggression in Ukraine, the occurrence of a NATO collective defense scenario in Eastern Europe with direct German involvement is being discussed as a possible scenario within the coming years.

In such a case, Germany would assume a central role in the medical and nursing care of wounded soldiers and civilians, leading to profound changes in the German healthcare system.

The preparedness and resilience of the German healthcare system in the context of such a crisis should be critically examined in light of the experiences from the COVID-19 pandemic. Since current students and trainees are the future providers of healthcare services, they represent a key factor in the resilience of the German healthcare system in the event of a crisis. Therefore, you are the focus of this survey.

The aim of this survey is to assess the **P**reparedness (Vorbereitung), **O**wnership (Identifikation), **W**illingness (Bereitschaft), and **A**wareness (Bewusstsein) of future healthcare workers in the medical, nursing, and paramedic fields with regard to a NATO collective defense scenario in Eastern Europe with German involvement, in order to better evaluate the resilience of the German healthcare system.

Participant Information:

The study is directed exclusively at **current trainees and students of healthcare professions** (nursing, paramedics, human medicine, health sciences, etc.).

The survey takes approximately 15 minutes. Participation in the survey is voluntary. No registration is required, and you may discontinue the survey at any time. Once the questionnaire has been submitted, withdrawal from the study is no longer possible due to the anonymization of your data.

The survey is conducted in cooperation with medical and public health students from various German universities under the leadership of Akkon University of Human Sciences. If you have any questions or comments, you may contact the study team at any time: powa-analysis@outlook.de

Further detailed information on the study's purpose, those responsible, the study procedure, the benefits and risks, the voluntariness of participation, and data protection and data use can be found [here].

Data Protection:

The following personal data will be collected: Age, sex, citizenship, residence permit, field of study/apprenticeship, study/apprenticeship progress, prior vocational training.

These data serve for further analysis and interpretation of the results. Your responses will be anonymized upon submission of the questionnaire, so that no conclusions can be drawn about your

identity. The collected data will not be analyzed on an individual basis, but only at an aggregate level.

The handling of your personal data is described in detail in the attached privacy policy.

Declaration of Consent:

By clicking “Yes” on the first page of the online survey, you agree that you:

- have taken note of the participant information and the attached privacy policy for this study,
- have been informed of the possibility of downloading the participant information, privacy policy, and this consent form,
- are sufficiently informed and had the opportunity to ask questions prior to continuing the study,
- consent to the processing of your personal data in the manner described above, and
- agree to participate in the study.

Thank you for your participation and your trust!
Your Project Team

EL02

I consent and wish to participate in the survey. I further agree to the publication of the survey results.

○ Yes

BA

BA01

1. What is your current field of study or vocational training?

○ Medicine (university degree program)
○ Nursing (university degree program)
○ Nursing apprenticeship (Gesundheitspflege/Krankenpflege)
○ Paramedic apprenticeship (Notfallsanitäter*in)
○ Other field of study with patient contact (Please enter title)
○ Other vocational training with patient contact (Please enter title)
○ Other field of study/vocational training

5 active filter(s)

Filter BA01/F1
If one of the following response option(s) was selected: **-1**
Then after clicking "Next", display text **VE01** and end the interview

Filter BA01/F2
If one of the following response option(s) was selected: **1, 2, 5**
Then display question/text **BA05** later in the questionnaire (otherwise hide)

Filter BA01/F3
If one of the following response option(s) was selected: **3, 4, 6**
Then display question/text **BA04** later in the questionnaire (otherwise hide)

Filter BA01/F4
If one of the following response option(s) was selected: **3, 4, 6**
Then hide question/text **BA05** later in the questionnaire

Filter BA01/F5
If one of the following response option(s) was selected: **3, 4, 6**
Then hide question/text **BA03** later in the questionnaire

BA02

2. Have you completed a degree program, vocational training, and/or basic military training with the German Armed Forces (Bundeswehr)?

○ Yes
○ No

BA07

3. How old are you?

[Please select]

1 active filter

Filter BA07/F1
If one of the following response option(s) was selected: **1, -9**
Then after clicking “Next”, display text **VE01** and end the interview

---

BA08

4. What gender do you identify with?

○ Female
○ Male
○ Diverse
○ Prefer not to say

BA09

5. What is your citizenship?

○ German citizenship
○ EU citizenship
○ Other citizenship with permanent residence in Germany
○ Other citizenship without permanent residence in Germany
○ German citizenship plus other citizenship

1 active filter

Filter BA09/F1
If one of the following response option(s) was selected: **4**
Then after clicking “Next”, display text **VE01** and end the interview

---

BA05

6. You have indicated that you are currently studying.
Have you completed vocational training prior to your studies?

○ Yes, nursing apprenticeship
○ Yes, paramedic apprenticeship
○ Yes, other vocational training (Please enter)
○ No

1 active filter

Filter BA05/F1
If one of the following response option(s) was selected: **1**
Then display question/text later in the questionnaire (otherwise hide)

BA04

7. You have indicated that you are currently completing a vocational apprenticeship.
In which year of your apprenticeship are you?

[Please select]

BA03

8. Please indicate your study progress.
Stated in subject semesters (total number of semesters enrolled in the current field of study)

[Please select]

EG 1

EG08

9. Have you engaged in voluntary/honorary work before or during your apprentice-ship/studies?

○ Yes
○ No

1 active filter

Filter EG08/F1
If one of the following response option(s) was selected: **1**
Then display page(s) **EG 2–EG 3** of the questionnaire (otherwise hide)

EG 2

EG09

10. Thinking exclusively about the last 3 years: Over what period of time have you engaged in voluntary/honorary work?

○ Not at all
○ For one year or less
○ Between one and two years
○ More than two years / over the entire period

EG01

11. How frequently have you carried out your voluntary or honorary engagement on average over the last three years?

○ Not at all
○ Less than once a month
○ Once a month
○ Several times a month
○ Once a week
○ Several times a week
○ Daily

EG06

12. How many hours per week do you engage in voluntary work on average?
Please estimate the number of hours per week.

[Please select]

EG03

13. Do you engage voluntarily in any of the following areas?
Multiple answers are possible.

☐ Aid organizations: DRK, ASB, JUH, DLRG or MHD
☐ Volunteer fire department
☐ Federal Agency for Technical Relief (THW)
☐ None of the above areas

1 active filter

Filter EG03/F1
If one of the following response option(s) was selected: **1, 2, 3**
Then display question/text **EG07** later in the questionnaire (otherwise hide)

EG 3

EG07

14. You have indicated that you are involved in an aid organization, the THW, or the volunteer fire department.

Are you of the opinion that this experience will prove helpful for your work in healthcare in the event of a military crisis situation?

○ Does not apply at all
○ Rather does not apply
○ Rather applies
○ Fully applies

PO1

PO24

This section concerns your political attitudes and convictions.

PO17

15. Which of the following reasons do you consider most likely to be the cause of the war between Russia and Ukraine?
Multiple answers are possible.

☐ Russia’s legitimate security interests (e.g. due to NATO’s eastward expansion)
☐ Russia’s legitimate interests based on ethnic and historical ties with Ukraine
☐ Russia’s internationally unlawful aggression against Ukraine
☐ Russia’s power and geopolitical interests (e.g. in the context of expanding Russia’s sphere of leadership and dominance)

PO18

16. What is your position on Germany spending at least 2% of its gross domestic product (GDP) on defense in accordance with the NATO defense spending guideline?

Defense spending of 2% of GDP is:

○ Too much
○ Rather too much
○ Appropriate
○ Rather too little
○ Too little

PO19

17. Are you of the opinion that compulsory military service should be reintroduced in Germany?

○ Strongly disagree
○ Rather disagree
○ Rather agree
○ Strongly agree

PO20

18. In Germany, many people tend to support a particular political party over a longer period of time, even if they do not always vote for it.

Do you – generally speaking – tend to favor a particular party represented in the last Bundestag, and if so, which one:

Listed in order of results from the 2021 Bundestag election

○ SPD
○ CDU/CSU
○ Bündnis 90 / Die Grünen (Green Party)
○ FDP
○ AFD
○ Die Linke (Left Party)
○ BSW
○ Other party
○ No party affiliation

3 active filter(s)

Filter PO20/F1
If one of the following response option(s) was selected: **1, 2, 3, 4, 5, 6, 7, 8**
Then display question/text **PO21** later in the questionnaire (otherwise hide)

Filter PO20/F2
If one of the following response option(s) was selected: **1, 2, 3, 4, 5, 6, 7, 8**
Then display question/text **PO22** later in the questionnaire (otherwise hide)

Filter PO20/F3
If one of the following response option(s) was selected: **1, 2, 3, 4, 5, 6, 7, 8**
Then display question/text **PO23** later in the questionnaire (otherwise hide)

PO2

PO23

You have indicated that you particularly favor a party represented in the Bundestag.

PO21

19. How strongly or weakly do you – all things considered – favor this party?

○ Very weakly
○ Rather weakly
○ Rather strongly
○ Very strongly

PO22

20. Do you favor this party in principle, or do you currently prefer this party because it seems more suitable to you in the present situation?

○ Fundamental affiliation with this party
○ Temporary preference for this party
○ Don’t know

RE 1

RE08

This section concerns your assessment of the likelihood and consequences of, and your attitude toward, a possible NATO collective defense scenario in Eastern Europe with German involvement.

RE01

21. How likely do you consider a NATO collective defense scenario in Eastern Europe with German involvement by 2030?

○ Not likely
○ Less likely
○ Rather likely
○ Very likely

RE02

22. To what extent does the prospect of a NATO collective defense scenario in Eastern Europe with German involvement cause you concern in your daily life?

○ No concern at all
○ Rather little concern
○ Rather great concern
○ Very great concern

RE03

23. To what extent do you consider a NATO collective defense scenario in Eastern Europe with German involvement to have negative effects on the German healthcare system?

○ No negative effects at all
○ Rather minor negative effects
○ Rather major negative effects
○ Very major negative effects

RE05

24. Are you of the opinion that healthcare workers have a special responsibility to contribute to the healthcare system beyond their normal duties (e.g. under changed working conditions and hours) in the event of a NATO collective defense scenario in Eastern Europe with German involvement?

○ Do not agree
○ Rather disagree
○ Rather agree
○ Fully agree

RE 2

RE09

This section concerns your attitude toward specific content in your apprenticeship/studies.

RE06

25. Are you of the opinion that disaster medicine should be a mandatory component of the curriculum in your apprenticeship/studies?

○ Do not agree
○ Rather disagree
○ Rather agree
○ Fully agree

RE07

26. Are you of the opinion that the sociopolitical implications of possible disaster medicine scenarios should also be addressed within your apprenticeship/studies (e.g. the significance of society-wide defense capability)?

○ Do not agree
○ Rather disagree
○ Rather agree
○ Fully agree

PE 1

PE15

This section captures your assessment of individual preparedness (Preparedness) for a possible NATO collective defense scenario in Eastern Europe with German involvement.

PE14

27. To what extent have the following topics been covered in your apprenticeship/studies?

Please indicate the highest level applicable to you. The examples of course content listed are intended to illustrate the “fundamentals” being asked about. Please do not base your assessment solely on whether each individual example topic has been covered.

Fundamentals of general medical care under disaster conditions (e.g. life-saving immediate measures, shock treatment, pain management and anesthesia)

○ No prior engagement
○ Addressed in a course
○ Actively practiced
○ Can apply independently

Fundamentals of specific medical care under disaster conditions (e.g. surgical emergency measures, treatment of thermal injuries, chemical and biological accidents, chemical warfare agents)

○ No prior engagement
○ Addressed in a course
○ Actively practiced
○ Can apply independently

Principles of hospital alarm and evacuation planning (e.g. for mass casualty incidents, infections, radioactive contamination, fire)

○ No prior engagement
○ Addressed in a course
○ Actively practiced
○ Can apply independently

Fundamentals of disaster-induced mental health conditions and measures for psychosocial support (e.g. acute stress response, post-traumatic stress disorder, in the context of psychosocial emergency care)

○ No prior engagement
○ Addressed in a course
○ Actively practiced
○ Can apply independently

PE12

28. Have you already attended a voluntary seminar/training/summer school on the topic of disaster medicine and humanitarian aid (e.g. organized by the Foundation of the German Institute for Disaster Medicine or the Institute for Rescue and Emergency Medicine), or have you already taken an elective university course in this area?

○ Yes
○ No

PE 2

PE13

This section concerns your assessment of the structural, i.e. health-system-level, preparedness for a possible NATO collective defense scenario in Eastern Europe with German involvement.

PE07

29. How do you assess the general current state of the German healthcare system?

○ Poor
○ Rather poor
○ Rather good
○ Good

PE08

30. How well do you consider the German healthcare system to be prepared for the expected additional burdens in the event of a possible NATO collective defense scenario in Eastern Europe with German involvement?

○ Poorly
○ Rather poorly
○ Rather well
○ Well

PE09

31. In your opinion, is the security of hospitals and medical facilities in Germany guaranteed in the event of a military conflict (e.g. protection from attacks on civilian infrastructure)?

○ Not guaranteed
○ Less guaranteed
○ Rather guaranteed
○ Guaranteed

BE1

BE07

This section concerns your willingness (Willingness) to adapt to new requirements and to engage beyond your normal duties in the context of your current studies/apprenticeship or at your future workplace, before and during possible disaster medicine scenarios.

BE09

32. Preventive education and training in the context of crisis and disaster medicine, as well as the practice of emergency plans, contributes to resilience in healthcare in the event of future crisis situations.

Can you currently imagine voluntarily…

… attending free events in the area of disaster and crisis medicine at your current training institution (vocational school/university/university of applied sciences)?

○ Yes
○ No

… participating in exercises for emergency and evacuation plans or crisis and emergency medicine staff training at your future workplace?

○ Yes
○ No

… participating in exercises for emergency and evacuation plans or crisis and emergency medicine staff training organized by external providers (professional societies, aid organizations)?

○ Yes
○ No

… independently initiating continuing education events and practical exercises in crisis and disaster medicine at your future workplace?

○ Yes
○ No

… becoming involved/continuing to be involved with a national aid organization (DRK, ASB, JHU, DLRG, MHD) in the future?

○ Yes
○ No

… obtaining further training within a voluntary role at a national aid organization in support of the Bundeswehr Medical Service?

○ Yes
○ No

… advocating for strengthening the cooperation between professional and voluntary actors in medical care?

○ Yes
○ No

… voluntarily registering in a registry through which you could be contacted in the event of a crisis when medical personnel are needed?

○ Yes
○ No

… acquiring relevant leadership skills in the crisis and disaster medicine context through further training?

○ Yes
○ No

BE2

BE10

33. In a NATO collective defense scenario in Eastern Europe with German involvement, Germany is said by experts to have a central role in medical and nursing care, which would lead to structural changes in the German healthcare system and altered working conditions.

Which of the following activities/scenarios are personally feasible for you?

Working outside your own area of specialization to maintain routine care (e.g. as a dermatologist treating minor injuries / instead of working in inpatient nursing, working in OR nursing)

○ Yes
○ I am not sure
○ No

Switching from outpatient to inpatient care

○ Yes
○ I am not sure
○ No

Performing triage (categorization of patients by severity of illness/injury)

○ Yes
○ I am not sure
○ No

Working beyond normal working hours, even if this brings you to your physical and psychological limits over an extended period of time

○ Yes
○ I am not sure
○ No

Leaving your profession in healthcare to avoid additional work burdens

○ Yes
○ I am not sure

○ No

Changing your place of work within Germany due to changed personnel needs to ensure patient care

○ Yes
○ I am not sure
○ No

Emigrating from Germany (e.g. to a neutral country such as Switzerland) for personal advantage

○ Yes
○ I am not sure
○ No

Taking on a leadership role to coordinate patient care

○ Yes
○ I am not sure
○ No

BE3

BE13

34. In a NATO collective defense scenario in Eastern Europe with German involvement, Germany is said by experts to have a central role in medical and nursing care, which would lead to structural changes in the German healthcare system.

Would you be willing to take on the following medical or nursing responsibilities?

Care of injured German civilians – in Germany

○ Yes
○ I am not sure
○ No

Care of wounded German soldiers – in Germany

○ Yes
○ I am not sure
○ No

Care of wounded soldiers from NATO allied partners – in Germany

○ Yes
○ I am not sure
○ No

Care of wounded German soldiers – abroad

○ Yes
○ I am not sure
○ No

Care of injured civilians of a NATO allied partner – abroad

○ Yes
○ I am not sure
○ No

Care of wounded soldiers of a NATO allied partner – abroad

○ Yes
○ I am not sure
○ No

Support of the Bundeswehr Medical Service – abroad

○ Yes
○ I am not sure
○ No

Working in a civilian healthcare facility of a NATO allied partner – abroad

○ Yes
○ I am not sure
○ No

Support of the Medical Service of a NATO allied partner – abroad

○ Yes
○ I am not sure
○ No

Conducting patient transport for the distribution of patients within NATO allied countries

○ Yes
○ I am not sure
○ No

BE4

BE14

35. Please rate possible motivations in terms of their personal importance for your engagement in healthcare in the event of a NATO collective defense scenario in Eastern Europe with German involvement.

1 = no importance, 10 = highest importance for engagement

Legal/occupational obligations

1 –––––––––––––––––––– 10
○ ○ ○ ○ ○ ○ ○ ○ ○ ○

Professional ethics

1 –––––––––––––––––––– 10
○ ○ ○ ○ ○ ○ ○ ○ ○ ○

Perceived relevance and meaningfulness of the issue

1 ––––––––––––––––––––– 10
○ ○ ○ ○ ○ ○ ○ ○ ○ ○

Social recognition

1 ––––––––––––––––––––– 10
○ ○ ○ ○ ○ ○ ○ ○ ○ ○

Societal expectations toward my professional group

1 ––––––––––––––––––––– 10
○ ○ ○ ○ ○ ○ ○ ○ ○ ○

Monetary compensation

1 ––––––––––––––––––––– 10
○ ○ ○ ○ ○ ○ ○ ○ ○ ○

Patriotism

1 ––––––––––––––––––––– 10
○ ○ ○ ○ ○ ○ ○ ○ ○ ○

Networking and exchange with other volunteers

1 ––––––––––––––––––––– 10
○ ○ ○ ○ ○ ○ ○ ○ ○ ○

Continuing education opportunities

1 ––––––––––––––––––––– 10
○ ○ ○ ○ ○ ○ ○ ○ ○ ○

BE08

36. From the main motivations listed above, select the three most important motivations for your engagement in healthcare in the event of a NATO collective defense scenario in Eastern Europe with German involvement.

Please do not select any motivation twice!

Most important motivation
[Please select]

Second most important motivation
[Please select]

Third most important motivation
[Please select]

Closing

By submitting through clicking the “Next” button, it is no longer possible to withdraw from the study. Your responses will then be saved anonymously.

Last Page

Thank you for your participation!

We sincerely thank you for your support.

Your responses have been saved; you may now close the browser window.

POWA-Analysis powa@akkon-hochschule.de
