# Supplementary material for: Preparedness, awareness and ownership of young future healthcare professionals are associated with their willingness to contribute to health system resilience in a NATO collective defense scenario: a cross-sectional study among medical, nursing and paramedic students in Germany
Source: BMC Public Health. 2026 Oct 2;26:2758. doi: 10.1186/s12889-026-29675-1 (PMC13632156; doi:10.1186/s12889-026-29675-1)
Supplement: Supplementary file 4 [file 12889_2026_29675_MOESM4_ESM.pdf]

Survey results - descriptive tables

List of tables

Table 1: Descriptive statistics of the metric variables
Table 2: Current field of study or vocational training
Table 3: Another degree programme involving work with patients
Table 4: Another VET programme involving work with patients
Table 5: Vocational training, and/or basic military training with the German Armed Forces (Bundeswehr)
Table 6: VET programme before studies
Table 7: Other prior vocational training
Table 8: Gender
Table 9: Citizenship
Table 10: Willingness Part I: Preventive education and training
Table 11: Willingness Part II: Willingness to contribute in the case of a NATO collective defense scenario in Eastern Europe
Table 12: Willingness Part III: Willingness to contribute in the case of a NATO collective defense scenario in Eastern Europe
Table 13: Motivation for engagement
Table 14: Main motives for engagement
Table 15: Voluntary engagement
Table 16: Frequency of voluntary engagement
Table 17: Years of voluntary engagement in the last 3 years [years]
Table 18: Do you engage voluntarily in any of the following areas
Table 19: Preparedness through engagement in aid organizations
Table 20: General state of the German healthcare system
Table 21: Preparedness of the German healthcare
Table 22: Security of hospitals and medical facilities in Germany in the event of a military conflict
Table 23: Topics covered in apprenticeship/studies
Table 24: Seminar/Training/Summer school on the topic of disaster medicine and humanitarian aid
Table 25: Cause of the war between Russia and Ukraine
Table 26: Germany's spending on defense
Table 27: Compulsory military service
Table 28: Party preference
Table 29: Party affiliation
Table 30: Do you favor this party in principle, or do you currently ...
Table 31: Likelihood of a NATO collective defense scenario
Table 32: Concern regarding a NATO collective defense scenario
Table 33: Negative effects a NATO collective defense scenario for Germany
Table 34: Special responsibility of healthcare workers in the event of a NATO collective defense scenario
Table 35: Disaster medicine in the curricula of apprenticeships/studies

Table 1: Descriptive statistics of the metric variables

Variable	Mean	SD	Median	IQR	Range	*N*
Study Progress	7.4	3.7	7.5	4.0 – 10.0	1.0 – 18.0	*330*
Progress in the VET program	1.9	0.9	2.0	1.0 – 3.0	1.0 – 4.0	*184*
Age [years]	24.5	4.5	24.0	21.0 – 26.0	18.0 – 36.0	*514*
Voluntary Engagement [hrs/week]	2.6	4.1	1.5	0.0 – 4.0	0.0 – 25.0	*514*
Years of voluntary engagement in the last 3 years [years]	1.3	1.1	1.5	0.0 – 2.5	0.0 – 2.5	*514*
Willingness Score [0-54]	36.4	9.9	38.0	30.0 – 44.0	0.0 – 54.0	*514*
Individual Preparedness Score [0-12]	3.3	2.6	3.0	1.0 – 5.0	0.0 – 12.0	*514*
Institutional Preparedness Score [0-9]	2.2	1.5	2.0	1.0 – 3.0	0.0 – 6.0	*514*
Awareness Score [0-63]	17.6	11.8	17.0	11.0 – 23.0	0.0 – 63.0	*514*
Awareness Score [log transformed]	2.7	0.8	2.9	2.5 – 3.2	0.0 – 4.2	*514*

Table 2: Current field of study or vocational training

Items	Medicine (university degree program)	Nursing (university degree program)	Nursing apprentice ship (Gesundhe itspflege/K rankenpfle ge)	Paramedic apprentice ship (Notfallsa nitäter*in)	Other field of study with patient contact (Please enter title)	Other vocational training with patient contact (Please enter title)	*N*
What is your current field of study or vocational training?	302 (59%)	18 (4%)	74 (14%)	102 (20%)	10 (2%)	8 (2%)	*514*

Table 3: Another degree programme involving work with patients

Response	n	%
Rescue services and emergency care	8	80%
Advanced Nursing Practice	1	10%
Work-study programme in applied nursing sciences	1	10%
**Total**	**10**	**100%**

Table 4: Another VET programme involving work with patients

Response	n	%
Generalist nursing apprenticeship	7	88%
Geriatric nursing assistant	1	12%
**Total**	**8**	**100%**

Table 5: Vocational training, and/or basic military training with the German Armed Forces (Bundeswehr)

Items	No	Yes	*N*
Have you completed a degree program, vocational training, and/or basic military training with the German Armed Forces (Bundeswehr)?	479 (93%)	35 (7%)	*514*

Table 6: VET programme before studies

Items	Yes, nursing apprenticeship	Yes, paramedic apprenticeship	Yes, other vocational training (Please enter)	*N*
VET programme before studies	21 (21%)	14 (14%)	65 (65%)	*100*

Table 7: Other prior vocational training

Response	n	%
Paramedic (Rettungssanitäter)	41	63%
Operating room technical assistant (OTA)	3	5%
Medical assistant (MFA)	2	3%
Nursing apprenticeship (started, not completed)	2	3%
Physiotherapy	2	3%
Biological technical assistant (BTA)	1	2%
Clinical study assistant	1	2%
Construction mechanic	1	2%
Degree in molecular medicine; paramedic	1	2%
Degree in political science	1	2%
Degree in Romance linguistics	1	2%
Emergency medical technician and operating room technical assistant	1	2%
Hospitality management	1	2%
Medical laboratory assistant (MTLA)	1	2%
Medical technical assistant for functional diagnostics (MTAF)	1	2%
Midwife; degree in health sciences	1	2%
Nursing assistant (Krankenpflegehelfer)	1	2%
Operating room technical assistant and emergency medical technician	1	2%
Paramedic (Rettungsassistent)	1	2%
Primary-qualifying dual nursing degree (state examination and bachelor's in eight semesters)	1	2%
**Total**	**65**	**100%**

Table 8: Gender

Items	Female	Male	*N*
What gender do you identify with?	293 (57%)	221 (43%)	*514*

Table 9: Citizenship

Items	German citizenship	EU citizenship	Other citizenship with permanent residence in Germany	German citizenship plus other citizenship	*N*
What is your citizenship?	467 (91%)	4 (1%)	8 (2%)	35 (7%)	*514*

Table 10: Willingness Part I: Preventive education and training

Can you currently imagine voluntarily …

Items	No	Yes	*N*
… attending free events in the area of disaster and crisis medicine at your current training institution (vocational school/university/university of applied sciences)?	44 (9%)	470 (91%)	*514*
… participating in exercises for emergency and evacuation plans or crisis and emergency medicine staff training at your future workplace?	24 (5%)	490 (95%)	*514*

Items	No	Yes	*N*
… participating in exercises for emergency and evacuation plans or crisis and emergency medicine staff training organized by external providers (professional societies, aid organizations)?	87 (17%)	427 (83%)	*514*
… independently initiating continuing education events and practical exercises in crisis and disaster medicine at your future workplace?	327 (64%)	187 (36%)	*514*
… becoming involved/continuing to be involved with a national aid organization (DRK, ASB, JHU, DLRG, MHD) in the future?	187 (36%)	327 (64%)	*514*
… obtaining further training within a voluntary role at a national aid organization in support of the Bundeswehr Medical Service?	282 (55%)	232 (45%)	*514*
… advocating for strengthening the cooperation between professional and voluntary actors in medical care?	126 (25%)	388 (75%)	*514*
… voluntarily registering in a registry through which you could be contacted in the event of a crisis when medical personnel are needed?	155 (30%)	359 (70%)	*514*
… acquiring relevant leadership skills in the crisis and disaster medicine context through further training?	113 (22%)	401 (78%)	*514*

Table 11: Willingness Part II: Willingness to contribute in the case of a NATO collective defense scenario in Eastern Europe

Would you be willing to take on the following medical or nursing responsibilities?

Items	No	I am not sure	Yes	*N*
Care of injured German civilians – in Germany	5 (1%)	18 (4%)	491 (96%)	*514*
Care of wounded German soldiers – in Germany	9 (2%)	26 (5%)	479 (93%)	*514*
Care of wounded soldiers from NATO allied partners – in Germany	13 (3%)	34 (7%)	467 (91%)	*514*
Care of wounded German soldiers – abroad	145 (28%)	180 (35%)	189 (37%)	*514*
Care of injured civilians of a NATO allied partner – abroad	136 (26%)	186 (36%)	192 (37%)	*514*
Care of wounded soldiers of a NATO allied partner – abroad	151 (29%)	183 (36%)	180 (35%)	*514*
Support of the Bundeswehr Medical Service – abroad	195 (38%)	178 (35%)	141 (27%)	*514*
Working in a civilian healthcare facility of a NATO allied partner – abroad	170 (33%)	186 (36%)	158 (31%)	*514*
Support of the Medical Service of a NATO allied partner – abroad	197 (38%)	191 (37%)	126 (25%)	*514*
Conducting patient transport for the distribution of patients within NATO allied countries	137 (27%)	149 (29%)	228 (44%)	*514*

Table 12: Willingness Part III: Willingness to contribute in the case of a NATO collective defense scenario in Eastern Europe

Which of the following activities/scenarios are personally feasible for you?

Items	No	I am not sure	Yes	*N*
Working outside your own area of specialization to maintain routine care (e.g. as a dermatologist treating minor injuries / instead of working in inpatient nursing, working in OR nursing)	48 (9%)	133 (26%)	333 (65%)	*514*
Switching from outpatient to inpatient care	75 (15%)	105 (20%)	334 (65%)	*514*
Performing triage (categorization of patients by severity of illness/injury)	33 (6%)	94 (18%)	387 (75%)	*514*
Working beyond normal working hours, even if this brings you to your physical and psychological limits over an extended period of time	92 (18%)	160 (31%)	262 (51%)	*514*
Leaving your profession in healthcare to avoid additional work burdens	282 (55%)	177 (34%)	55 (11%)	*514*
Changing your place of work within Germany due to changed personnel needs to ensure patient care	202 (39%)	223 (43%)	89 (17%)	*514*
Emigrating from Germany (e.g. to a neutral country such as Switzerland) for personal advantage	175 (34%)	191 (37%)	148 (29%)	*514*
Taking on a leadership role to coordinate patient care	70 (14%)	167 (32%)	277 (54%)	*514*

Table 13: Motivation for engagement

Please rate possible motivations in terms of their personal importance for engagement (1 = no importance, 10 = highest importance)

Items	1	2	3	4	5	6	7	8	9	10	*N*
Legal/occupational obligations	50 (10%)	30 (6%)	59 (11%)	46 (9%)	79 (15%)	71 (14%)	68 (13%)	55 (11%)	24 (5%)	32 (6%)	*514*
Professional ethics	17 (3%)	4 (1%)	11 (2%)	14 (3%)	32 (6%)	39 (8%)	66 (13%)	97 (19%)	106 (21%)	128 (25%)	*514*
Perceived relevance and meaningfulness of the issue	20 (4%)	6 (1%)	10 (2%)	10 (2%)	38 (7%)	32 (6%)	64 (12%)	100 (19%)	102 (20%)	132 (26%)	*514*
Social recognition	66 (13%)	41 (8%)	82 (16%)	56 (11%)	72 (14%)	53 (10%)	51 (10%)	45 (9%)	16 (3%)	32 (6%)	*514*
Societal expectations toward my professional group	49 (10%)	24 (5%)	44 (9%)	42 (8%)	59 (11%)	65 (13%)	77 (15%)	59 (11%)	46 (9%)	49 (10%)	*514*
Monetary compensation	90 (18%)	47 (9%)	66 (13%)	55 (11%)	68 (13%)	58 (11%)	42 (8%)	33 (6%)	14 (3%)	41 (8%)	*514*
Patriotism	189 (37%)	52 (10%)	40 (8%)	47 (9%)	45 (9%)	45 (9%)	29 (6%)	30 (6%)	16 (3%)	21 (4%)	*514*
Networking and exchange with other volunteers	89 (17%)	44 (9%)	59 (11%)	56 (11%)	66 (13%)	53 (10%)	48 (9%)	41 (8%)	23 (4%)	35 (7%)	*514*
Continuing education opportunities	45 (9%)	20 (4%)	36 (7%)	43 (8%)	54 (11%)	48 (9%)	62 (12%)	78 (15%)	44 (9%)	84 (16%)	*514*

Table 14: Main motives for engagement

Response	***Most important motivation***	***Second most important motivation***	***Third most important motivation***
Legal/occupational obligations	31 (6%)	52 (10%)	66 (13%)
Professional ethics	253 (49%)	108 (21%)	32 (6%)
Perceived relevance and meaningfulness of the issue	12 (2%)	32 (6%)	27 (5%)
Social recognition	12 (2%)	56 (11%)	92 (18%)
Societal expectations toward my professional group	28 (5%)	22 (4%)	49 (10%)
Monetary compensation	11 (2%)	23 (4%)	22 (4%)
Patriotism	11 (2%)	18 (4%)	51 (10%)
Networking and exchange with other volunteers	33 (6%)	71 (14%)	102 (20%)
Continuing education opportunities	123 (24%)	132 (26%)	73 (14%)
N	*514*	*514*	*514*

Table 15: Voluntary engagement

Have you engaged in voluntary/honorary work …

Items	No	Yes	*N*
… before or during your apprenticeship/studies?	142 (28%)	372 (72%)	*514*
… in the last three years?	210 (41%)	304 (59%)	*514*

Table 16: Frequency of voluntary engagement

Items	Daily	Several times a week	Once a week	Several times a month	Once a month	Less than once a month	*N*
How frequently have you carried out your voluntary or honorary engagement on average over the last three years?	13 (4%)	86 (28%)	46 (15%)	94 (31%)	44 (14%)	21 (7%)	*304*

Table 17: Years of voluntary engagement in the last 3 years [years]

Items	For one year or less	Between one and two years	More than two years / over the entire period	*N*
Years of voluntary engagement in the last 3 years [years]	42 (14%)	52 (17%)	210 (69%)	*304*

Table 18: Do you engage voluntarily in any of the following areas

Multiple answers are possible.

Items	Not selected	Selected	*N*
Aid organizations: DRK, ASB, JUH, DLRG or MHD	200 (66%)	104 (34%)	*304*
Volunteer fire department	253 (83%)	51 (17%)	*304*
Federal Agency for Technical Relief (THW)	298 (98%)	6 (2%)	*304*

Table 19: Preparedness through engagement in aid organizations

Items	Does not apply at all	Rather does not apply	Rather applies	Fully applies	*N*
You have indicated that you are involved in an aid organization, the THW, or the volunteer fire department. Are you of the opinion that this experience will prove helpful for your work in healthcare in the event of a military crisis situation?	7 (5%)	15 (11%)	49 (37%)	62 (47%)	*133*

Table 20: General state of the German healthcare system

Items	Poor	Rather poor	Rather good	Good	*N*
How do you assess the general current state of the German healthcare system?	107 (21%)	252 (49%)	145 (28%)	10 (2%)	*514*

Table 21: Preparedness of the German healthcare

Items	Poorly	Rather poorly	Rather well	Well	*N*
How well do you consider the German healthcare system to be prepared for the expected additional burdens in the event of a possible NATO collective defense scenario in Eastern Europe with German involvement?	286 (56%)	199 (39%)	28 (5%)	1 (0%)	*514*

Table 22: Security of hospitals and medical facilities in Germany in the event of a military conflict

Items	Not guaranteed	Less guaranteed	Rather guaranteed	Guaranteed	*N*
In your opinion, is the security of hospitals and medical facilities in Germany guaranteed in the event of a military conflict (e.g. protection from attacks on civilian infrastructure)?	255 (50%)	216 (42%)	42 (8%)	1 (0%)	*514*

Table 23: Topics covered in apprenticeship/studies

To what extent have the following topics been covered in your apprenticeship/studies? Please indicate the highest level applicable to you. The examples of course content listed are intended to illustrate the "fundamentals" being asked about. Please do not base your assessment solely on whether each individual example topic has been covered.

Items	No prior engagement	Addressed in a course	Actively practiced	Can apply independently	*N*
Fundamentals of general medical care under disaster conditions (e.g. life-saving immediate measures, shock treatment, pain management and anesthesia)	119 (23%)	131 (25%)	146 (28%)	118 (23%)	*514*
Fundamentals of specific medical care under disaster conditions (e.g. surgical emergency measures, treatment of thermal injuries, chemical and biological accidents, chemical warfare agents)	280 (54%)	179 (35%)	32 (6%)	23 (4%)	*514*
Principles of hospital alarm and evacuation planning (e.g. for mass casualty incidents, infections, radioactive contamination, fire)	354 (69%)	124 (24%)	15 (3%)	21 (4%)	*514*
Fundamentals of disaster-induced mental health conditions and measures for psychosocial support (e.g. acute stress response, post-traumatic stress disorder, in the context of psychosocial emergency care)	209 (41%)	243 (47%)	39 (8%)	23 (4%)	*514*

Table 24: Seminar/Training/Summer school on the topic of disaster medicine and humanitarian aid

Items	No	Yes	*N*
Have you already attended a voluntary seminar/training/summer school on the topic of disaster medicine and humanitarian aid (e.g. organized by the Foundation of the German Institute for Disaster Medicine or the Institute for Rescue and Emergency Medicine), or have you already taken an elective university course in this area?	443 (86%)	71 (14%)	*514*

Table 25: Cause of the war between Russia and Ukraine

Which of the following reasons do you consider most likely to be the cause of the war between Russia and Ukraine? Multiple answers are possible.

Items	Not selected	Selected	*N*
Russia's legitimate security interests (e.g. due to NATO's eastward expansion)	457 (89%)	57 (11%)	*514*
Russia's legitimate interests based on ethnic and historical ties with Ukraine	493 (96%)	21 (4%)	*514*
Russia's internationally unlawful aggression against Ukraine	151 (29%)	363 (71%)	*514*
Russia's power and geopolitical interests (e.g. in the context of expanding Russia's sphere of	57 (11%)	457 (89%)	*514*

Table 26: Germany's spending on defense

Items	Too little	Rather too little	Appropriate	Rather too much	Too much	*N*
What is your position on Germany spending at least 2% of its gross domestic product (GDP) on defense in accordance with the NATO defense spending guideline?	41 (8%)	97 (19%)	250 (49%)	70 (14%)	56 (11%)	*514*

Table 27: Compulsory military service

Items	Strongly agree	Rather agree	Rather disagree	Strongly disagree	*N*
Are you of the opinion that compulsory military service should be reintroduced in Germany?	76 (15%)	148 (29%)	139 (27%)	151 (29%)	*514*

Table 28: Party preference

Items	Bündnis 90 / Die Grünen (Green Party)	CDU/CSU	SPD	Die Linke (Left Party)	FDP	AFD	BSW	Other party	Not stated	*N*
In Germany, many people tend to support a particular political party over a longer period of time, even if they do not always vote for it. Do you – generally speaking – tend to favor a particular party represented in the last Bundestag, and if so, which one:	178 (35%)	48 (9%)	41 (8%)	95 (18%)	11 (2%)	23 (4%)	10 (2%)	15 (3%)	93 (18%)	*514*

Table 29: Party affiliation

Items	Very weakly	Rather weakly	Rather strongly	Very strongly	*N*
How strongly or weakly do you – all things considered – favor this party?	7 (2%)	93 (22%)	282 (67%)	39 (9%)	*421*

Table 30: Do you favor this party in principle, or do you currently ...

Items	Fundamental affiliation with this party	Temporary preference for this party	*N*
Do you favor this party in principle, or do you currently prefer this party because it seems more suitable to you in the present situation?	313 (78%)	88 (22%)	*401*

Table 31: Likelihood of a NATO collective defense scenario

Items	Not likely	Less likely	Rather likely	Very likely	*N*
How likely do you consider a NATO collective defense scenario in Eastern Europe with German involvement by 2030?	42 (8%)	221 (43%)	214 (42%)	37 (7%)	*514*

Table 32: Concern regarding a NATO collective defense scenario

Items	No concern at all	Rather little concern	Rather great concern	Very great concern	*N*
To what extent does the prospect of a NATO collective defense scenario in Eastern Europe with German involvement cause you concern in your daily life?	47 (9%)	211 (41%)	181 (35%)	75 (15%)	*514*

Table 33: Negative effects a NATO collective defense scenario for Germany

Items	No negative effects at all	Rather minor negative effects	Rather major negative effects	Very major negative effects	*N*
To what extent do you consider a NATO collective defense scenario in Eastern Europe with German involvement to have negative effects on the German healthcare system?	3 (1%)	83 (16%)	259 (50%)	169 (33%)	*514*

Table 34: Special responsibility of healthcare workers in the event of a NATO collective defense scenario

Items	Do not agree	Rather disagree	Rather agree	Fully agree	*N*
Are you of the opinion that healthcare workers have a special responsibility to contribute to the healthcare system beyond their normal duties (e.g. under changed working conditions and hours) in the event of a NATO collective defense scenario in Eastern Europe with German involvement?	56 (11%)	112 (22%)	222 (43%)	124 (24%)	*514*

Table 35: Disaster medicine in the curricula of apprenticeships/studies

This section concerns your attitude toward specific content in your apprenticeship/studies.

Items	Do not agree	Rather disagree	Rather agree	Fully agree	*N*
Are you of the opinion that disaster medicine should be a mandatory component of the curriculum in your apprenticeship/studies?	13 (3%)	49 (10%)	196 (38%)	256 (50%)	*514*
Are you of the opinion that the sociopolitical implications of possible disaster medicine scenarios should also be addressed within your apprenticeship/studies (e.g. the significance of society-wide defense capability)?	37 (7%)	117 (23%)	214 (42%)	146 (28%)	*514*
